# Estimates of Alpha/Beta (α/β) Ratios for Individual Late Rectal Toxicity Endpoints: An Analysis of the CHHiP Trial

**DOI:** 10.1016/j.ijrobp.2020.12.041

**Published:** 2021-06-01

**Authors:** Douglas H. Brand, Sarah C. Brüningk, Anna Wilkins, Katie Fernandez, Olivia Naismith, Annie Gao, Isabel Syndikus, David P. Dearnaley, Alison C. Tree, Nicholas van As, Emma Hall, Sarah Gulliford, CHHiP Trial Management Group

**Affiliations:** ∗ Division of Radiotherapy and Imaging, The Institute of Cancer Research, London, United Kingdom; † Urology Unit, Royal Marsden NHS Foundation Trust, London, United Kingdom; ‡ Department of Biosystems Science and Engineering, ETH Zurich, Basel, Switzerland; § Tumour Cell Biology Laboratory, The Francis Crick Institute, London, United Kingdom; ‖ Radiotherapy Trials QA Group, Royal Marsden NHS Foundation Trust, London, United Kingdom; ¶ Radiotherapy Department, Clatterbridge Cancer Centre, United Kingdom; # Clinical Trials and Statistics Unit, The Institute of Cancer Research, London, United Kingdom; ∗∗ Department of Medical Physics and Biomedical Engineering, University College London, London, United Kingdom; †† Department of Radiotherapy Physics, University College London Hospitals NHS Foundation Trust, London, United Kingdom

## Abstract

**Purpose:**

Changes in fraction size of external beam radiation therapy exert nonlinear effects on subsequent toxicity. Commonly described by the linear-quadratic model, fraction size sensitivity of normal tissues is expressed by the α/β ratio. We sought to study individual α/β ratios for different late rectal effects after prostate external beam radiation therapy.

**Methods and Materials:**

The CHHiP trial (ISRCTN97182923) randomized men with nonmetastatic prostate cancer 1:1:1 to 74 Gy/37 fractions (Fr), 60 Gy/20 Fr, or 57 Gy/19 Fr. Patients in the study had full dosimetric data and zero baseline toxicity. Toxicity scales were amalgamated to 6 bowel endpoints: bleeding, diarrhea, pain, proctitis, sphincter control, and stricture. Lyman-Kutcher-Burman models with or without equivalent dose in 2 Gy/Fr correction were log-likelihood fitted by endpoint, estimating α/β ratios. The α/β ratio estimate sensitivity was assessed using sequential inclusion of dose modifying factors (DMFs): age, diabetes, hypertension, inflammatory bowel or diverticular disease (IBD/diverticular), and hemorrhoids. 95% confidence intervals (CIs) were bootstrapped. Likelihood ratio testing of 632 estimator log-likelihoods compared the models.

**Results:**

Late rectal α/β ratio estimates (without DMF) ranged from bleeding (G1 + α/β = 1.6 Gy; 95% CI, 0.9-2.5 Gy) to sphincter control (G1 + α/β = 3.1 Gy; 95% CI, 1.4-9.1 Gy). Bowel pain modelled poorly (α/β, 3.6 Gy; 95% CI, 0.0-840 Gy). Inclusion of IBD/diverticular disease as a DMF significantly improved fits for stool frequency G2+ (*P* = .00041) and proctitis G1+ (*P* = .00046). However, the α/β ratios were similar in these no-DMF versus DMF models for both stool frequency G2+ (α/β 2.7 Gy vs 2.5 Gy) and proctitis G1+ (α/β 2.7 Gy vs 2.6 Gy). Frequency-weighted averaging of endpoint α/β ratios produced: G1 + α/β ratio = 2.4 Gy; G2 + α/β ratio = 2.3 Gy.

**Conclusions:**

We estimated α/β ratios for several common late adverse effects of rectal radiation therapy. When comparing dose-fractionation schedules, we suggest using late a rectal α/β ratio ≤ 3 Gy.

## Introduction

Moderately hypofractionated external beam radiation therapy (EBRT) for the curative treatment of nonmetastatic prostate cancer (PCa) has gained broad acceptance following reports of efficacy and safety from the CHHiP, PROFIT, and RTOG 0415 hypofractionation studies.^1^, ^2^, ^3^ Each trial randomized between moderately hypofractionated and conventional dose-escalated EBRT regimens, and all showed noninferiority of the hypofractionated regimens for 5-year biochemical and clinical progression-free survival. A fourth study, HYPRO, unfortunately failed to establish superiority of a dose-escalated, hypofractionated schedule, which demonstrated increased toxicity.^4^

Rectal toxicity endpoints are important late adverse effects of prostate EBRT. Models have been produced for many common individual rectal endpoints, such as bleeding, proctitis, stool frequency, and fecal incontinence.^5^, ^6^, ^7^, ^8^, ^9^, ^10^, ^11^ These models incorporate dose-volume histogram (DVH)–derived values as dosimetric predictors. In the hypofractionation era, researchers have adjusted the rectal dose bins using the linear-quadratic model,^12^ describing normal tissue fraction sensitivity by means of the α/β ratio. Commonly, a late rectal α/β = 3 Gy is assumed^13^^,^^14^ to produce equivalent dose in 2 Gy fractions (EQD2) and to enable comparison with standard 2 Gy/fraction treatments.^12^ Similarly, EQD2 correction has been used when summating brachytherapy and EBRT doses, with α/β = 3 to 5.4 Gy.^15^, ^16^, ^17^

These EQD2-corrected comparisons of regimens are dependent on an accurate estimate of the α/β ratio. Researchers have previously provided human estimates for the α/β ratio of overall late rectal toxicity in the range 2.7 to 7.2 Gy.^18^, ^19^, ^20^, ^21^ However, individual rectal toxicity endpoints (eg, bleeding, urgency) are driven by different upstream pathophysiologic processes^22^ and may thus have distinct sensitivity to fraction size, as manifest by the α/β ratio. Although individual endpoint estimates have been produced for the central nervous system,^23^ to our knowledge, such estimates have not previously been made for pelvic normal tissues.

Using data from a phase 3 trial of hypofractionated radiation therapy (RT), this study aims to estimate α/β ratios for individual rectal toxicity endpoints: bleeding, stool frequency, proctitis, sphincter control, and stricture or ulcer. It also aims to test whether such α/β ratio estimates are influenced by inclusion of other predictive clinical factors: age, diabetes, hypertension, inflammatory bowel disease (IBD) or diverticular disease, and hemorrhoids.

## Methods and Materials

### The CHHiP trial

The CHHiP trial (ISRCTN97182923) has previously been described in detail.^1^^,^^24^^,^^25^ Briefly, 3216 men were recruited, all with histologically confirmed T1b–T3aN0M0 prostate adenocarcinoma, prostate specific antigen ≤40 ng/mL and risk of lymph node involvement <30%. Open-label randomization was performed 1:1:1 between conventional (74 Gy in 37 fractions [Fr] over 7.4 weeks), higher dose hypofractionated (60 Gy in 20 Fr over 4 weeks) or lower dose hypofractionated (57 Gy in 19 Fr over 3.8 weeks) EBRT. The primary endpoint of biochemical or clinical failure was met, with noninferiority of the 60 Gy/20 Fr regimen confirmed.^1^ Ethics approval has been described previously.^1^ The Institute of Cancer Research Clinical Trials and Statistics Unit (ICR-CTSU, London, UK) coordinated the study and managed the data used in this analysis.

### Patient cohort and Digital Imaging and Communications in Medicine files

CHHiP trial patients who received all fractions of one of the protocol RT regimens were eligible for inclusion in this substudy. Those without centrally available Digital Imaging and Communications in Medicine (DICOM) data from computed tomography, structures, and dose cube were excluded. Non-DICOM treatment plan file types were converted to DICOM.

### Rectal contouring and dose-volume histogram generation

The CHHiP trial protocol recommended, ideally, an empty rectum. Contouring for the rectum, as a solid structure, was “from the anus (usually at the level of the ischial tuberosities or 1 cm below the lower margin of the PTV whichever is more inferior) to the recto-sigmoid junction.”^1^ Quality assurance (ie, adherence to the CHHiP trial protocol specifications of rectal contour) was undertaken for the contoured rectums on all DICOM data sets obtained, by 1 of 5 trained observers. In particular, attention was paid to the inferior and superior extent of contour. Once the rectal contour was checked, and recontoured where necessary, the rectal DVH was recalculated for use in this study.

### Endpoints

The CHHiP trial collected bowel toxicity information in the form of both clinician-reported outcomes^1^ and patient-reported outcomes (PROs).^25^ Clinician-reported outcomes were chosen, because PRO measures changed during the course of the trial. These sources were Radiation Therapy Oncology Group (RTOG) late rectal toxicity,^26^ the Royal Marsden Hospital (RMH) scale,^27^ and Late Effects Normal Tissue – Subjective, Objective & Management (LENT-SOM).^28^ Only the Royal Marsden Hospital and LENT-SOM data were collected at registration (baseline) and before RT. All scales were collected for late rectal toxicity at 6-, 12-, 18-, 24-, 36-, 48-, 60-month follow-up after the start of RT.

The scales were merged into new amalgamated endpoints representing underlying separate symptomatic issues, using a methodology described previously.^29^ Grading was simplified to grade 0 for no toxicity, grade 1 for toxicity not needing intervention, and grade 2 for any toxicity requiring intervention. The scores were dichotomized to consider: grade 0 versus grade 1 and grade 2 or greater (G1 + comparison); grade 0 and grade 1 versus grade 2 or greater (G2 + comparison). For bowel pain, sphincter control and stricture/ulcer, grade ≥2 events were rare (<5%); therefore, only a G1 + comparison was performed. No attempt was made to amalgamate endpoints to generate G3+ models, both owing to the rarity of G3+ events and the difficulty of unifying such events between scales.

For each endpoint, patients were excluded if any relevant toxicity was reported at baseline or before RT assessments, or if both assessments were missing. The point of this exclusion was to avoid those with pre-existing symptoms registering as having treatment-induced toxicity events during follow-up. Patients were further excluded for an endpoint if they were missing the relevant follow-up data at more than 3 of the 7 (>50%) late toxicity assessments. Toxicity events were scored for any relevant toxicity of sufficient grade at any time point (ie, worst toxicity). A full description of the endpoint generation process is provided in Appendix E1.

### Generalized Lyman-Kutcher-Burman model

A generalized Lyman-Kutcher-Burman (LKB) model has been described previously for rectal α/β ratio estimation.^20^ Dose modifying factors (DMFs) were incorporated as modulators of each individual patient’s effective dose parameter (D_Eff_), per prior work by Tucker et al.^30^ The model is expressed as a definite integral:

(1) $$NTCP=\frac{1}{\sqrt{2\pi }}\cdot \int_{-\infty }^{t}e^{-0.5\cdot x^{2}}dx\;$$

where *NTCP* is the normal tissue complication probability. Furthermore:

(2) $$t=\frac{D_{Eff}\cdot e^{\delta \cdot DMF}-TD_{50}}{m\cdot TD_{50}}\;$$

Here, *TD*_*50*_ represents the tolerance dose for 50% toxicity, at the median (steepest) part of the NTCP dose response curve; *m* is a parameter inversely controlling the steepness at *TD*_*50*_*. DMF* is the dose modifying factor corresponding to either: ones and zeros for binary risk factors, or a positive integer for age; *δ* is the dose modifying coefficient, used to adjust *TD*_*50*_ in the presence of the risk factor specified by *DMF.* For binary DMFs, the coefficient is for presence of risk factor; for numerical DMFs (age only), it is evaluated on a per-unit basis. Note that a DMF covariate of zero will result in no change to the effective dose (*D*_*Eff*_), which is defined by:

(3) $$D_{Eff}={\left(\sum_{i=1}^{z}{\left(EQD2_{i}\right)}^{\frac{1}{n}}\cdot v_{i}\right)}^{n}\;$$

where *n* represents the relative seriality of a tissue endpoint dose response, with values toward 0 being more serial and toward 1 being more parallel^31^; *z* is the number of dose bins, iterated by dose bin *i*; and *v*_*i*_ is the relative volume of an organ present in the dose bin *i. EQD2*_*i*_*,* is the EQD2 for dose bin *i,* which is given by:

(4) $$EQD2_{i}=D_{i}\cdot \left(\frac{d_{i}+\alpha /\beta }{2\;Gy+\alpha /\beta }\right)\;$$

where *D*_*i*_ is the total dose in Gy, to a given DVH dose bin *i*; *d*_*i*_ is the dose in Gy per fraction, to a given dose bin (ie, D_i_ divided by number of fractions); and *α/β* (Gy) is the theoretical single fraction dose giving equal contribution for linear (*α*) and quadratic (*β*) components of the linear-quadratic formula.^12^

This model is termed LKB-EQD2, or LKB-EQD2-DMF with the inclusion of a DMF in Equation 2. The LKB-NoEQD2 model without EQD2 correction uses Equations 1 and 2 (without DMF inclusion), but substitutes physical dose bin dose for *EQD2*_*i*_ in Equation 3. This LKB-NoEQD2 model was fitted separately for patients receiving 2 Gy per fraction (74 Gy in 37 Fr) and 3 Gy per fraction patients (60 Gy in 20 Fr and 57 Gy in 19 Fr).

### Initial grid search

For each model, initial fitting was done using the grid search method, as described previously.^7^ Each unknown parameter was searched on a grid with dimensionality equal to number-of-fit parameters (Table E1). LKB-EQD2 models with fixed α/β were also produced, using the same parameter grid as those with fitted α/β, but fixing the α/β to 3 or 4.8 Gy, per prior estimates.^19^^,^^20^

Model performance was assessed in 2 ways. First, the naive performance was assessed by calculating a log likelihood sum. Better model performance will produce a less negative log likelihood sum. It was calculated as:

(5) $$Likelihood=f\left(toxicity\right)=\left\{ \begin{array}{l} NTCP\;toxicity=1\\ 1-NTCP\;toxicity=0 \end{array}\right.\;$$

(6) $$Performance=Summed\;Log\;Likelihood=\sum_{j=1}^{c}\ln\;Likelihood_{j}\;$$

where *c* = number of patients (with *j* as iterator through such patients).

The model parameter values generating the ten least negative performance metrics were recorded at the end of the grid search. The best (least negative) of these was noted as the naive model performance for later use in Equation 8.

The second action at each grid step was to assess performance of 2000 bootstraps, drawn with replacement, with unique bootstraps for each endpoint. The bootstrap performance was also assessed with Equation 6. At the end of the grid search, the parameters giving the 10 least negative performance metrics for each bootstrap were recorded. The parameters resulting in best bootstrap performance were noted, so that these could be used later for out-of-the-bag prediction in Equation 7.^32^

### Second-stage search

To account for the known sensitivity of fitting algorithms to initial starting parameters and hence to improve model performance,^33^ a secondary optimization search for parameter values was undertaken. The values of *n*, *m*, *TD50*, *α/β*, and *DMFs* producing the 10 best performance metrics (by Equation 6) were used as the initial parameters in a constrained Nelder-Mead simplex algorithm search^34^ to determine whether further improvement in performance could be found (ie, for each endpoint): 1 naive model and 2000 bootstraps with 10 searches = 20010 algorithm searches. This algorithm was run with constraints: *n* = 0.01 to 10; *m* = 0.01 to 10; *TD50* = 0.01 to 1000 Gy. Where freely fitted, α/β was searched in space 0.001 to 1000 Gy. The dose modifying factor covariate was searched in space –10 to 10, which when raised to the natural base *e,* searches a dose multiplier range of 4.54 × 10^–4^ to 22,026. This wide bounding of all fit parameters was chosen to prevent bootstrap distributions being inappropriately constrained, which would bias the coverage of the nonparametric 95% confidence interval. For the naive likelihood and each bootstrap, the final best model parameters were those resulting in best performance (by Equation 6) from any of the grid search positions or any of the subsequent 10 Nelder-Mead simplex algorithm searches.

### Estimating test performance and model comparison

A model comprising more free parameters is always likely to improve naive likelihood performance, but this can be due to overfitting.^35^ To address this difficulty, the 632 bootstrap estimator was used as an unbiased estimator of test performance.^36^ It balances out the overoptimistic naive likelihood (fitted on the population) against the negatively biased out-of-the-bag bootstrap estimate. We preferred 632 over the 632+ bootstrap estimator, owing to faster calculation and the low risk of near-perfect prediction with a relatively simple model.^32^ The first step calculated the out-of-the-bag (OOB) performance for the model:

(7) $$OOB\;performance=\sum_{j=1}^{c}\left(\frac{1}{z}\times \sum_{boot=1}^{z}\ln{\hat{likelihood}}_{p,boot}\right)\;$$

where *c* is the total number of patients (iterated by *j*), and *z* is the number of bootstraps not containing patient *j* (iterated by *boot*). The predicted likelihood is derived by inserting the predicted NTCP into Equation 5.

The 632 estimator was then calculated^32^:

(8) 632 Estimator=0.368⋅Naive Performance+0.632⋅OOB Performance 

Models were compared by means of the likelihood ratio test of the 632 estimators. First, comparing whether the LKB-EQD2 model with free-fitted α/β ratio had significantly better 632 estimator than the model with the α/β ratio fixed at two reported literature values: α/β = 3 Gy or 4.8 Gy.^19^^,^^20^ Second, examining for significant improvement from LKB-EQD2 to LKB-EQD2-DMF, which was sequentially tested with each of the DMFs. Tests were planned only where log likelihood improvement occurred; with approximately 50 tests anticipated, a penalized *P* value of .001 was used for interpretation of significance.^37^ Parameter estimates were obtained at the 50th centile of the bootstrap distribution; 95% bootstrap confidence intervals (CIs) for the optimal model parameter values were obtained as the 2.5th and 97.5th centiles of the corresponding parameter values producing the best summed log likelihood performance metric for each bootstrap.

### Graphical outputs of calibration

Model calibration was fitted as a logistic regression of predicted NTCP values for each patient as a single predictor against observed binary outcomes (toxicity or no toxicity). The fitted model was then displayed graphically against ideal (perfect) prediction—termed the *calibration curve*. Furthermore, binned calibration plots were examined, with patients grouped into deciles of predicted risk: average bin NTCP plotted against observed bin toxicity proportion.

### Software

Processing of trial data into the endpoints used for this study was done with Stata (version 15; Statacorp). VODCA (version 5.4.1; Medical Software Solutions) was used to convert non-DICOM data to DICOM and for the checking of rectal contours. MATLAB (version 2018b; MathWorks) was used to import DVH data from DICOM files and for all modeling using custom scripts. Nelder-Mead simplex algorithm searches were performed with a modified bounded version of *fminsearch* (fminsearchbnd, version 1.4.0.0).^38^ Tables were formatted in Excel 2019 and Word 2019 (Microsoft). All plots were produced in MATLAB.

## Results

**Fig. 1:**
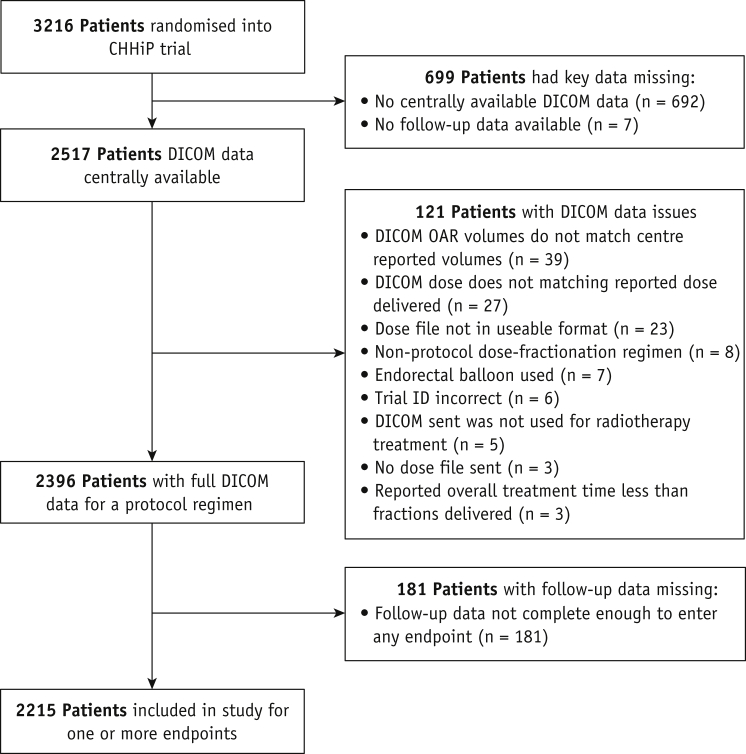
Patient flow diagram showing any reasons for exclusion of all patients originally randomized into the CHHiP trial. *Abbreviations:* DICOM = Digital Imaging and Communications in Medicine; ID = Identity; OAR = organ at risk.

Two thousand two hundred fifteen patients from the CHHiP trial had appropriate data for this analysis. Figure 1 is a CONSORT-style flow diagram accounting for all patients who were originally randomized into the CHHiP study and their reasons for noninclusion in this analysis. Key relevant baseline and treatment characteristics for the included patients are shown in Table 1, which are similar to those in the CHHiP trial as a whole. These date indicate that patients in this study are representative of the whole trial cohort. The cumulative rectal DVH curves for all patients, separated by fractionation arm, are shown in Appendix E2. A summary of the number of patients meeting requirements (≥50% follow-up form completion) for each endpoint modelled is shown in Table 2, with the proportion of patients expressing toxicity ranging from 3.6% for stricture/ulcer G1+ (79/2206) to 38.1% for stool frequency G1+ (771/2025). The influence of excluding patients with baseline toxicity on categorical DMF proportions is examined in Table E2. For some endpoints, patients with DMF present were overrepresented in those excluded for baseline toxicity versus those included in study: IBD/diverticular disease and both rectal bleeding G1+ and G2+; pelvic surgery and stricture/ulcer G1+; hemorrhoids and rectal bleeding G1+ and G2+, frequency G1+ and G2+, pain G1+, proctitis G1+ and G2+.

**Table 1:** Baseline characteristics for patients∗

Characteristic	This Study†		Whole CHHiP Trial‡	
	n	%	n	%
Arm				
57 Gy/19 fractions	755	34	1077	33
60 Gy/20 fractions	753	34	1074	33
74 Gy/37 fractions	707	32	1065	33
NCCN risk group				
Low	308	14	484	15
Intermediate	1655	75	2347	73
High	252	11	385	12
Gleason score				
≤6	750	34	1122	35
7	1399	63	1995	62
8	66	3	99	3
Clinical T stage				
T1	851	38	1170	36
T2	1196	54	1766	55
T3	167	8	277	9
Missing	1	<1	3	<1
Pre-ADT PSA				
<10 ng/mL	1082	49	1567	49
10-20 ng/mL	1006	45	1415	44
≥20 ng/mL	127	6	208	6
Missing	0	0	26	<1
Comorbidities				
Diabetes	227	10	342	11
Hypertension	874	40	1276	40
IBD or diverticular disease	85	4	124	4
Pelvic surgery	162	7	252	8
Symptomatic hemorrhoids	153	7	209	6
Total	2215	100	3216	100

*Abbreviations:* ADT = androgen deprivation therapy; IBD = Inflammatory Bowel Disease; NCCN = National Comprehensive Cancer Network; PSA = prostate specific antigen.

∗ Hypertension included even if medically controlled.

† Median age, 69 years (range, 44-85 years).

‡ Median age, 69 years (range, 44-85 years).

**Table 2:** Summary of patient numbers in each modeling endpoint∗

Rectal endpoints and grades of interest	Dose fractionation regimen						Total	
	57 Gy in 19 fractions		60 Gy in 20 fractions		74 Gy in 37 fractions			
	n	%	n	%	n	%	n	%
Bleeding G1+								
No	479	70.5	434	63.4	434	67.4	1347	67.1
Yes	200	29.5	251	36.6	210	32.6	661	32.9
Excluded	73		67		67		207	
Bleeding G2+								
No	590	86.9	575	83.9	549	85.5	1714	85.4
Yes	89	13.1	110	16.1	93	14.5	292	14.6
Excluded	73		67		69		209	
Frequency G1+								
No	437	62.8	428	62.4	389	60.5	1254	61.9
Yes	259	37.2	258	37.6	254	39.5	771	38.1
Excluded	56		66		68		190	
Frequency G2+								
No	611	87.9	587	85.8	545	84.9	1743	86.2
Yes	84	12.1	97	14.2	97	15.1	278	13.8
Excluded	57		68		69		194	
Pain G1+								
No	686	93.1	671	90.1	638	90.8	1995	91.3
Yes	51	6.9	74	9.9	65	9.2	190	8.7
Excluded	15		7		8		30	
Proctitis G1+								
No	509	69.3	449	62.2	433	62.7	1391	64.8
Yes	225	30.7	273	37.8	258	37.3	756	35.2
Missing	18		30		20		68	
Proctitis G2+								
No	666	90.9	641	88.8	607	87.8	1914	89.2
Yes	67	9.1	81	11.2	84	12.2	232	10.8
Excluded	19		30		20		69	
Sphincter control G1+								
No	680	91.0	664	88.7	615	87.5	1959	89.1
Yes	67	9.0	85	11.3	88	12.5	240	10.9
Excluded	5		3		8		16	
Stricture/ulcer G1+								
No	732	97.5	719	95.9	676	95.9	2127	96.4
Yes	19	2.5	31	4.1	29	4.1	79	3.6
Excluded	1		2		6		9	
Total	752	100	752	100	711	100	2215	100

∗ Patients excluded for any of: missing baseline data; baseline toxicity grade >0; missing >50% of follow-up forms. Presented percentages are calculated without the inclusion of patients excluded for each endpoint, so that event rates in modeled patients can be seen.

Table 3 (upper 2 sections) shows parameter estimates of *n*, *m*, and *TD50* for fits of the LKB-NoEQD2 model to 2 groups: 74 Gy only or 57 and 60 Gy combined. Each endpoint is presented separately. Table 3 then shows LKB-EQD2 model fits for all patients combined, across the same endpoints, including estimates for the α/β ratio. We note that the α/β ratio estimates for most endpoints were <3 Gy, with the upper bound of the 95% CI for rectal bleeding G1+ being <3 Gy. The 95% CI for pain G1+ was extremely wide (α/β = 0.0-840 Gy), suggesting a poor fit for this endpoint (ie, limited dose dependency). Table 3 also shows fits for the LKB-EQD2 model, with an α/β ratio fixed at 3 and 4.8 Gy. The *P* values for likelihood ratio test comparison between the LKB-EQD2 model (unfixed α/β) and the 2 fixed α/β models are shown. In many cases, the less flexible model (LKB-EQD2 with fixed α/β ratio) had a better fit (by 632 estimator), implying overfitting and making likelihood ratio testing inappropriate. The LKB-EQD2 model with free α/β ratio was significantly better than the model with fixed α/β 4.8 Gy for rectal bleeding G1+ (*P* = .00032). Other comparisons, in which the LKB-EQD2 model with fitted α/β ratio was better, did not meet the adjusted significance threshold.

**Table 3:** Parameters for LKB-NoEQD2 model and LKB-EQD2 model∗

Model	Patients (N)	n (95% CI)	n (95% CI)	TD50 (95% CI), Gy	α/β Ratio, Gy	632 Likelihood	*P* value vs LKB-EQD2
LKB-NoEQD2 (74 Gy)							
Bleeding G1+	644	0.26 (0.01-1.12)	0.33 (0.09-0.68)	61.5 (54.5-74.0)	N/A	–401.8	N/A
Bleeding G2+	642	0.13 (0.01-0.42)	0.21 (0.06-0.43)	74.0 (67.2-96.6)	N/A	–262.6	N/A
Frequency G1+	643	0.17 (0.01-0.53)	0.30 (0.09-0.76)	60.8 (53.7-72.8)	N/A	–427.7	N/A
Frequency G2+	642	0.11 (0.03-0.69)	0.20 (0.09-0.49)	73.8 (66.2-98.6)	N/A	–269.9	N/A
Pain G1+	703	0.24 (0.01-3.15)	0.33 (0.15-0.61)	92.7 (72.2-271.6)	N/A	–216.5	N/A
Proctitis G1+	691	0.10 (0.01-0.18)	0.22 (0.08-0.50)	64.9 (60.8-73.7)	N/A	–452.2	N/A
Proctitis G2+	691	0.05 (0.01-0.14)	0.14 (0.06-0.44)	78.0 (71.6-111.6)	N/A	–254.3	N/A
Sphincter control G1+	703	0.19 (0.09-3.30)	0.29 (0.16-0.63)	81.7 (68.5-185.3)	N/A	–263.8	N/A
Stricture/ulcer G1+	705	0.28 (0.01-5.79)	0.16 (0.05-0.31)	74.4 (66.2-92.8)	N/A	–117.6	N/A
LKB-NoEQD2 (57 Gy/60 Gy)							
Bleeding G1+	1364	0.13 (0.07-0.20)	0.22 (0.15-0.31)	50.7 (48.2-53.8)	N/A	–845.9	N/A
Bleeding G2+	1364	0.11 (0.01-0.28)	0.22 (0.13-0.40)	61.7 (56.3-74.2)	N/A	–560.6	N/A
Frequency G1+	1382	0.20 (0.12-0.33)	0.47 (0.30-0.89)	50.5 (46.8-59.2)	N/A	–908.2	N/A
Frequency G2+	1379	0.26 (0.02-0.73)	0.33 (0.20-0.53)	64.9 (56.5-94.4)	N/A	–531.9	N/A
Pain G1+	1482	0.02 (0.01-9.99)	0.37 (0.16-0.69)	105.4 (69.5-619.1)	N/A	–429.8	N/A
Proctitis G1+	1456	0.09 (0.01-0.17)	0.34 (0.18-0.70)	56.5 (52.0-67.8)	N/A	–931.3	N/A
Proctitis G2+	1455	0.12 (0.01-4.16)	0.28 (0.15-0.58)	73.8 (61.6-153.8)	N/A	–477.8	N/A
Sphincter control G1+	1496	0.17 (0.09-0.29)	0.26 (0.17-0.43)	65.8 (58.0-93.9)	N/A	–486.6	N/A
Stricture/ulcer G1+	1501	0.17 (0.01-0.47)	0.20 (0.09-0.35)	72.3 (60.6-113.6)	N/A	–217.4	N/A
LKB-EQD2 (all patients)							
Bleeding G1+	2008	0.21 (0.08-0.34)	0.33 (0.20-0.47)	58.8 (54.2-66.0)	1.6 (0.9-2.5)	–1248.1	N/A
Bleeding G2+	2006	0.16 (0.01-0.34)	0.27 (0.14-0.42)	75.8 (68.2-88.6)	1.7 (0.7-3.0)	–822.6	N/A
Frequency G1+	2025	0.27 (0.17-0.44)	0.55 (0.39-0.86)	56.0 (51.4-62.3)	2.3 (0.9-5.3)	–1334.7	N/A
Frequency G2+	2021	0.31 (0.10-0.71)	0.36 (0.23-0.52)	75.7 (66.2-96.8)	2.7 (0.9-8.5)	–801.3	N/A
Pain G1+	2185	0.15 (0.01-9.89)	0.48 (0.21-0.68)	139.7 (88.7-499.1)	3.6 (0.0-839.6)	–647.4	N/A
Proctitis G1+	2147	0.14 (0.02-0.22)	0.42 (0.22-0.68)	63.6 (58.7-75.5)	2.7 (1.5-5.4)	–1384.1	N/A
Proctitis G2+	2146	0.11 (0.01-0.25)	0.30 (0.17-0.51)	87.8 (75.2-137.0)	2.7 (1.3-15.1)	–731.9	N/A
Sphincter control G1+	2199	0.23 (0.15-0.38)	0.32 (0.24-0.45)	79.3 (69.8-103.3)	3.1 (1.4-9.1)	–749.7	N/A
Stricture/ulcer G1+	2206	0.31 (0.01-0.74)	0.25 (0.10-0.34)	83.8 (71.5-110.3)	2.5 (0.9-8.2)	–335.1	N/A
LKB-EQD2 (all patients); fixed α/β = 3 Gy							
Bleeding G1+	2008	0.23 (0.15-0.35)	0.37 (0.28-0.51)	57.3 (53.5-61.8)	3.0 (3.0-3.0)	–1250.2	.042
Bleeding G2+	2006	0.19 (0.03-0.36)	0.32 (0.21-0.46)	75.8 (67.8-92.3)	3.0 (3.0-3.0)	–822.9	.49
Frequency G1+	2025	0.27 (0.17-0.42)	0.56 (0.40-0.86)	55.7 (51.5-62.2)	3.0 (3.0-3.0)	–1334	Better fit
Frequency G2+	2021	0.31 (0.10-0.71)	0.36 (0.25-0.52)	75.8 (66.3-97.4)	3.0 (3.0-3.0)	–800.3	Better fit
Pain G1+	2185	0.17 (0.01-9.98)	0.49 (0.24-0.70)	142.6 (89.4-701.6)	3.0 (3.0-3.0)	–646.6	Better fit
Proctitis G1+	2147	0.14 (0.02-0.22)	0.43 (0.25-0.68)	63.4 (58.6-75.6)	3.0 (3.0-3.0)	–1383.2	Better fit
Proctitis G2+	2146	0.12 (0.01-0.25)	0.30 (0.18-0.51)	88.1 (75.3-136.5)	3.0 (3.0-3.0)	–730.8	Better fit
Sphincter control G1+	2199	0.24 (0.15-0.38)	0.32 (0.24-0.45)	79.1 (69.9-103.4)	3.0 (3.0-3.0)	–748.7	Better fit
Stricture/ulcer G1+	2206	0.32 (0.01-0.74)	0.25 (0.13-0.35)	84.4 (71.7-115.0)	3.0 (3.0-3.0)	–334.2	Better fit
LKB-EQD2 (all patients); fixed α/β = 4.8 Gy							
Bleeding G1+	2008	0.28 (0.20-0.42)	0.46 (0.36-0.63)	57.0 (53.1-62.5)	4.8 (4.8-4.8)	–1254.6	.00032†
Bleeding G2+	2006	0.24 (0.14-0.46)	0.39 (0.30-0.54)	80.0 (69.5-105.9)	4.8 (4.8-4.8)	–824.9	.032
Frequency G1+	2025	0.29 (0.19-0.45)	0.63 (0.46-0.96)	55.6 (51.2-63.0)	4.8 (4.8-4.8)	–1335.2	.34
Frequency G2+	2021	0.34 (0.16-0.75)	0.40 (0.30-0.54)	77.5 (67.0-103.5)	4.8 (4.8-4.8)	–800.7	Better fit
Pain G1+	2185	0.21 (0.01-9.97)	0.52 (0.30-0.70)	152.5 (93.6-745.7)	4.8 (4.8-4.8)	–646.4	Better fit
Proctitis G1+	2147	0.16 (0.09-0.24)	0.52 (0.38-0.81)	63.3 (58.2-74.1)	4.8 (4.8-4.8)	–1383.8	Better fit
Proctitis G2+	2146	0.14 (0.02-0.27)	0.36 (0.25-0.54)	93.4 (77.7-148.5)	4.8 (4.8-4.8)	–731	Better fit
Sphincter control G1+	2199	0.24 (0.16-0.38)	0.34 (0.27-0.47)	81.3 (71.1-106.6)	4.8 (4.8-4.8)	–749	Better fit
Stricture/ulcer G1+	2206	0.36 (0.15-0.84)	0.28 (0.21-0.37)	87.5 (73.2-127.9)	4.8 (4.8-4.8)	–334.2	Better fit

*Abbreviations:* CI = confidence interval; G1+ = grade 1 or above; G2+ = grade 2 or above; LKB-EQD2 = Lyman-Kutcher-Burman model with equivalent dose in 2-Gy correction; LKB-NoEQD2 = Lyman-Kutcher-Burman model with no equivalent dose in 2-Gy correction; Pts = patients; N/A = Not Applicable (to model).

∗ The first 2 sections show LKB-NoEQD2 model fitted for each endpoint to the conventionally fractionated (74 Gy) patients and the hypofractionated (57 and 60 Gy) patients. The next 3 sections show the LKB-EQD2 model fitted with a varying α/β ratio, then fixed to α/β = 3 Gy and α/β = 4.8 Gy. *P* values are from likelihood ratio tests between an endpoint 632 likelihood in the fixed α/β LKB-EQD2 models and the same endpoint 632 likelihood in the unfixed LKB-EQD2 model. Note that “better fit” implies that the simpler fixed α/β ratio model has better (less negative) 632 estimator than the more complex model (varying α/β ratio), implying the more complex model is overfitted and making likelihood ratio testing inappropriate.

† Significant at adjusted *P* < .001.

The effect on model parameters of sequential inclusion of each DMF is reported in Table 4. For each endpoint, the LKB-EQD2 model results without inclusion of DMF are reproduced in the first row for ease of comparison. Where the goodness of fit (as assessed with the 632 estimator) was improved with inclusion of DMF, *P* values for likelihood ratio testing of the LKB-EQD2-DMF model against the LKB-EQD2 model are presented. Only 2 LKB-EQD2-DMF models improved on LKB-EQD2, by adjusted significance: IBD/diverticular disease for both stool frequency G2+ (DMF = 1.37; 95% CI, 1.13-1.82; *P* = .00041) and proctitis G1+ (DMF = 1.27; 95% CI, 1.10-1.58; *P* = .00046). In both cases, α/β ratio estimates of the LKB-EQD2 versus LKB-EQD2-DMF fits did not differ by a clinically relevant margin: stool frequency G2+ (2.7 vs 2.5 Gy), proctitis G1+ (2.7 vs 2.6 Gy). Although inclusion of other DMFs did not meet adjusted significance for model fit improvement, it can be seen in Table 4 that any differences between LKB-EQD2-DMF model and LKB-EQD2 model α/β ratio estimates are not clinically meaningful.

**Table 4:** Effects of dose modifying factor inclusion

Rectal endpoints and dose modifying factors	Patients (N)	*n* covariate	*m* covariate	TD50 covariate (Gy_EQD2_)	α/β ratio (Gy)	Dose-modifying factor covariate	632 Likelihood	Likelihood ratio *P* value
Bleeding G1+								
LKB-EQD2 (no DMF)	2008	0.21 (0.08-0.34)	0.33 (0.20-0.47)	58.8 (54.2-66.0)	1.6 (0.9-2.5)	N/A	–1248.1	N/A
Age (years)	2008	0.21 (0.08-0.35)	0.33 (0.21-0.47)	51.0 (36.0-68.9)	1.6 (0.9-2.5)	0.9976 (0.9937-1.0016)	–1248.3	Worse fit
Diabetes Y/N	2008	0.20 (0.08-0.34)	0.32 (0.20-0.47)	58.6 (54.0-66.1)	1.6 (0.9-2.5)	0.96 (0.87-1.03)	–1248.3	Worse fit
Hemorrhoids Y/N	2008	0.21 (0.09-0.35)	0.33 (0.21-0.47)	58.9 (54.3-66.1)	1.6 (0.9-2.5)	1.07 (0.96-1.20)	–1248.3	Worse fit
Hypertension Y/N	2008	0.21 (0.09-0.35)	0.33 (0.21-0.47)	58.4 (53.7-65.8)	1.6 (0.9-2.5)	0.98 (0.93-1.03)	–1248.8	Worse fit
IBD/diverticular Y/N	2008	0.21 (0.10-0.35)	0.33 (0.21-0.46)	58.9 (54.3-65.0)	1.6 (0.9-2.5)	1.13 (1.01-1.30)	–1246.8	.11
Pelvic surgery Y/N	2008	0.20 (0.08-0.34)	0.33 (0.21-0.47)	59.3 (54.5-66.7)	1.6 (0.9-2.5)	1.08 (1.00-1.18)	–1247.3	.21
Bleeding G2+								
LKB-EQD2 (no DMF)	2006	0.16 (0.01-0.34)	0.27 (0.14-0.42)	75.8 (68.2-88.6)	1.7 (0.7-3.0)	N/A	–822.6	N/A
Age (years)	2006	0.16 (0.01-0.36)	0.27 (0.14-0.44)	81.0 (57.0-124.3)	1.7 (0.7-3.0)	1.0004 (0.9956-1.0055)	–823.5	Worse fit
Diabetes Y/N	2006	0.16 (0.01-0.35)	0.27 (0.14-0.42)	75.4 (67.7-88.5)	1.7 (0.7-3.0)	0.94 (0.80-1.03)	–822.6	.91
Hemorrhoids Y/N	2006	0.16 (0.01-0.34)	0.27 (0.14-0.42)	76.1 (68.2-89.6)	1.7 (0.7-3.1)	1.11 (0.99-1.33)	–821.9	.21
Hypertension Y/N	2006	0.16 (0.01-0.33)	0.27 (0.14-0.42)	74.6 (66.9-87.5)	1.7 (0.7-3.0)	0.96 (0.89-1.01)	–822.2	.36
IBD/diverticular Y/N	2006	0.17 (0.01-0.36)	0.28 (0.14-0.42)	75.9 (68.3-90.1)	1.7 (0.7-3.0)	1.17 (1.03-1.44)	–820.2	.026
Pelvic surgery Y/N	2006	0.16 (0.01-0.35)	0.27 (0.14-0.42)	76.2 (68.3-89.3)	1.7 (0.7-3.1)	1.04 (0.94-1.16)	–823.2	Worse fit
Stool frequency G1+								
LKB-EQD2 (No DMF)	2025	0.27 (0.17-0.44)	0.55 (0.39-0.86)	56.0 (51.4-62.3)	2.3 (0.9-5.3)	N/A	–1334.7	N/A
Age (years)	2025	0.27 (0.17-0.44)	0.54 (0.39-0.81)	38.8 (30.0-57.9)	2.3 (0.9-5.3)	0.9942 (0.9903-1.0003)	–1334	.25
Diabetes Y/N	2025	0.27 (0.17-0.43)	0.55 (0.39-0.83)	56.6 (51.7-63.3)	2.3 (0.9-5.3)	1.09 (0.97-1.25)	–1334.5	.52
Hemorrhoids Y/N	2025	0.28 (0.17-0.45)	0.56 (0.40-0.88)	56.8 (51.9-63.3)	2.2 (0.8-5.1)	1.21 (1.06-1.48)	–1331.8	.016
Hypertension Y/N	2025	0.27 (0.17-0.44)	0.55 (0.39-0.86)	55.6 (50.9-62.4)	2.2 (0.8-5.2)	0.98 (0.89-1.06)	–1335.5	Worse fit
IBD/diverticular Y/N	2025	0.27 (0.17-0.44)	0.55 (0.39-0.84)	56.4 (51.4-62.9)	2.3 (0.9-5.5)	1.19 (1.00-1.47)	–1334	.23
Pelvic surgery Y/N	2025	0.26 (0.16-0.42)	0.56 (0.40-0.85)	56.8 (51.8-63.7)	2.3 (1.0-5.6)	1.13 (0.99-1.33)	–1334.1	.28
Stool frequency G2+								
LKB-EQD2 (no DMF)	2021	0.31 (0.10-0.71)	0.36 (0.23-0.52)	75.7 (66.2-96.8)	2.7 (0.9-8.5)	N/A	–801.3	N/A
Age (years)	2021	0.31 (0.11-0.73)	0.35 (0.24-0.50)	54.4 (30.0-90.0)	2.7 (0.9-8.2)	0.9947 (0.9852-1.0026)	–801.4	Worse fit
Diabetes Y/N	2021	0.31 (0.10-0.70)	0.36 (0.24-0.51)	75.7 (66.2-93.9)	2.6 (0.9-8.7)	1.02 (0.86-1.17)	–802.2	Worse fit
Hemorrhoids Y/N	2021	0.31 (0.10-0.71)	0.36 (0.24-0.51)	76.6 (66.6-95.0)	2.7 (1.0-8.9)	1.15 (0.98-1.40)	–800.6	.22
Hypertension Y/N	2021	0.31 (0.10-0.73)	0.36 (0.23-0.51)	75.2 (65.7-91.8)	2.6 (0.9-8.2)	0.97 (0.86-1.07)	–802.1	Worse fit
IBD/diverticular Y/N	2021	0.31 (0.10-0.68)	0.36 (0.23-0.50)	76.2 (66.5-95.3)	2.5 (0.8-7.1)	1.37 (1.13-1.82)	–795.1	.00041∗
Pelvic surgery Y/N	2021	0.31 (0.09-0.73)	0.36 (0.24-0.51)	76.7 (66.6-96.3)	2.7 (1.0-9.8)	1.11 (0.95-1.33)	–801.2	.71
Bowel pain G1+								
LKB-EQD2 (no DMF)	2185	0.15 (0.01-9.89)	0.48 (0.21-0.68)	139.7 (88.7-499.1)	3.6 (0.0-839.6)	N/A	–647.4	N/A
Age (years)	2185	0.15 (0.01-1.74)	0.50 (0.25-0.74)	87.0 (42.0-179.4)	5.0 (0.2-39.4)	0.9911 (0.4328-1.0064)	–647.9	Worse fit
Diabetes Y/N	2185	0.16 (0.01-9.79)	0.48 (0.21-0.68)	138.0 (88.0-522.4)	3.7 (0.0-838.7)	0.95 (0.05-1.83)	–648.3	Worse fit
Hemorrhoids Y/N	2185	0.16 (0.01-9.89)	0.48 (0.21-0.69)	142.5 (88.8-606.3)	3.9 (0.0-921.1)	1.26 (0.85-4.47)	–647.2	.54
Hypertension Y/N	2185	0.14 (0.01-9.97)	0.46 (0.21-0.68)	137.5 (89.0-591.5)	3.5 (0.0-951.1)	1.04 (0.69-2.07)	–648.2	Worse fit
IBD/diverticular Y/N	2185	0.31 (0.01-9.95)	0.52 (0.21-0.70)	151.1 (89.6-867.0)	3.3 (0.0-942.9)	1.79 (1.07-13.76)	–644.2	.011
Pelvic surgery Y/N	2185	0.19 (0.01-9.90)	0.49 (0.21-0.69)	142.2 (88.8-647.6)	4.1 (0.0-945.8)	1.06 (0.31-3.28)	–648.2	Worse fit
Proctitis G1+								
LKB-EQD2 (No DMF)	2147	0.14 (0.02-0.22)	0.42 (0.22-0.68)	63.6 (58.7-75.5)	2.7 (1.5-5.4)	N/A	–1384.1	N/A
Age (years)	2147	0.14 (0.02-0.22)	0.42 (0.22-0.68)	54.2 (36.0-79.8)	2.7 (1.5-5.4)	0.9975 (0.9912-1.0030)	–1384.6	Worse fit
Diabetes Y/N	2147	0.14 (0.02-0.23)	0.42 (0.21-0.68)	62.8 (57.8-74.2)	2.6 (1.5-5.3)	0.84 (0.65-0.94)	–1379	.0013
Hemorrhoids Y/N	2147	0.14 (0.02-0.22)	0.43 (0.22-0.69)	64.1 (59.3-75.2)	2.7 (1.6-6.0)	1.12 (1.01-1.32)	–1382.6	.081
Hypertension Y/N	2147	0.14 (0.02-0.21)	0.42 (0.21-0.68)	62.9 (57.8-74.4)	2.6 (1.5-5.2)	0.97 (0.90-1.02)	–1384.3	Worse fit
IBD/diverticular Y/N	2147	0.14 (0.02-0.22)	0.43 (0.22-0.68)	64.0 (59.3-75.1)	2.6 (1.5-5.4)	1.27 (1.10-1.58)	–1378	.00046∗
Pelvic surgery Y/N	2147	0.14 (0.02-0.21)	0.43 (0.23-0.70)	65.1 (59.6-76.6)	2.7 (1.6-6.2)	1.15 (1.04-1.38)	–1381	.012
Proctitis G2+								
LKB-EQD2 (no DMF)	2146	0.11 (0.01-0.25)	0.30 (0.17-0.51)	87.8 (75.2-137.0)	2.7 (1.3-15.1)	N/A	–731.9	N/A
Age (years)	2146	0.12 (0.02-0.26)	0.30 (0.16-0.49)	90.1 (75.0-252.8)	2.7 (1.2-9.0)	1.0021 (0.9966-1.0129)	–732	Worse fit
Diabetes Y/N	2146	0.11 (0.01-0.26)	0.30 (0.17-0.50)	86.9 (74.7-131.7)	2.7 (1.3-12.6)	0.90 (0.62-1.01)	–731.4	.31
Hemorrhoids Y/N	2146	0.11 (0.01-0.27)	0.30 (0.17-0.51)	88.1 (75.3-136.6)	2.7 (1.3-14.6)	1.06 (0.92-1.31)	–732.2	Worse fit
Hypertension Y/N	2146	0.11 (0.01-0.29)	0.30 (0.17-0.49)	86.7 (74.6-125.7)	2.6 (1.2-9.4)	0.96 (0.84-1.03)	–732.2	Worse fit
IBD/diverticular Y/N	2146	0.11 (0.01-0.26)	0.30 (0.17-0.51)	88.8 (75.5-138.6)	2.6 (1.2-11.2)	1.22 (1.04-1.69)	–728.9	.015
Pelvic surgery Y/N	2146	0.11 (0.01-0.28)	0.30 (0.17-0.51)	89.2 (75.9-142.2)	2.8 (1.3-14.7)	1.11 (0.99-1.42)	–730.9	.16
Sphincter control G1+								
LKB-EQD2 (no DMF)	2199	0.23 (0.15-0.38)	0.32 (0.24-0.45)	79.3 (69.8-103.3)	3.1 (1.4-9.1)	N/A	–749.7	N/A
Age (years)	2199	0.24 (0.15-0.38)	0.34 (0.24-0.45)	90.0 (63.0-186.1)	3.0 (1.4-8.5)	1.0024 (0.9968-1.0102)	–750	Worse fit
Diabetes Y/N	2199	0.24 (0.15-0.39)	0.32 (0.24-0.45)	78.8 (69.4-99.7)	3.1 (1.4-9.4)	0.93 (0.73-1.06)	–750.2	Worse fit
Hemorrhoids Y/N	2199	0.24 (0.15-0.38)	0.32 (0.24-0.44)	80.3 (70.2-104.1)	3.2 (1.5-10.2)	1.15 (1.00-1.37)	–748.5	.14
Hypertension Y/N	2199	0.24 (0.15-0.38)	0.32 (0.24-0.42)	79.4 (69.7-95.4)	3.1 (1.4-8.8)	1.01 (0.93-1.10)	–750.5	Worse fit
IBD/diverticular Y/N	2199	0.24 (0.15-0.40)	0.33 (0.24-0.45)	80.6 (70.2-104.0)	3.1 (1.4-8.8)	1.29 (1.10-1.64)	–745.3	.0032
Pelvic surgery Y/N	2199	0.24 (0.15-0.39)	0.33 (0.24-0.45)	80.5 (70.2-103.6)	3.2 (1.4-10.2)	1.11 (0.96-1.30)	–749.4	.48
Stricture/ulcer G1+								
LKB-EQD2 (no DMF)	2206	0.31 (0.01-0.74)	0.25 (0.10-0.34)	83.8 (71.5-110.3)	2.5 (0.9-8.2)	N/A	–335.1	N/A
Age (years)	2206	0.28 (0.01-0.63)	0.25 (0.15-0.31)	136.4 (78.7-343.7)	2.4 (0.9-6.7)	1.0071 (0.9990-1.0184)	–333.9	.12
Diabetes Y/N	2206	0.31 (0.01-0.74)	0.25 (0.11-0.34)	83.6 (71.4-110.0)	2.5 (0.9-8.1)	0.97 (0.74-1.12)	–336.1	Worse fit
Hemorrhoids Y/N	2206	0.31 (0.01-0.75)	0.25 (0.11-0.34)	83.8 (71.6-109.4)	2.5 (0.9-8.2)	1.04 (0.84-1.23)	–336	Worse fit
Hypertension Y/N	2206	0.31 (0.01-0.74)	0.24 (0.11-0.33)	84.5 (71.9-108.3)	2.5 (0.9-7.5)	1.03 (0.93-1.13)	–335.8	Worse fit
IBD/diverticular Y/N	2206	0.32 (0.01-0.76)	0.25 (0.12-0.35)	84.1 (71.6-112.7)	2.5 (0.9-8.5)	1.05 (0.73-1.33)	–336.3	Worse fit
Pelvic surgery Y/N	2206	0.32 (0.01-0.76)	0.25 (0.11-0.35)	85.0 (72.0-113.8)	2.6 (1.0-9.4)	1.08 (0.91-1.30)	–335.5	Worse fit

Abbreviations: DMF = Dose Modifying Factor; LKB-EQD2 (No DMF) = Lyman-Kutcher Burman model with No DMF; 95% CI = 95% confidence interval; LKB-EQD2-DMF = Lyman-Kutcher Burman model with Equivalent Dose in 2Gy correction and DMF inclusion; Pts = patients; G1+ = grade 1 or above; G2+ = grade 2 or above; IBD = Inflammatory Bowel Disease.

Model fits for the sequential inclusion of each dose modifying factor, including the 632 estimator for model performance. Each DMF model is compared against the LKB-EQD2 (no DMF) model for the same endpoint by likelihood ratio test. Note that “worse fit” implies that the more complicated LKB-EQD2-DMF has a worse 632 estimator fit than the simpler LKB-EQD2 (no DMF) model, implying overfitting and making likelihood ratio testing inappropriate.

∗ Bold *P* values are significant at adjusted *P* < .001.

**Fig. 2:**
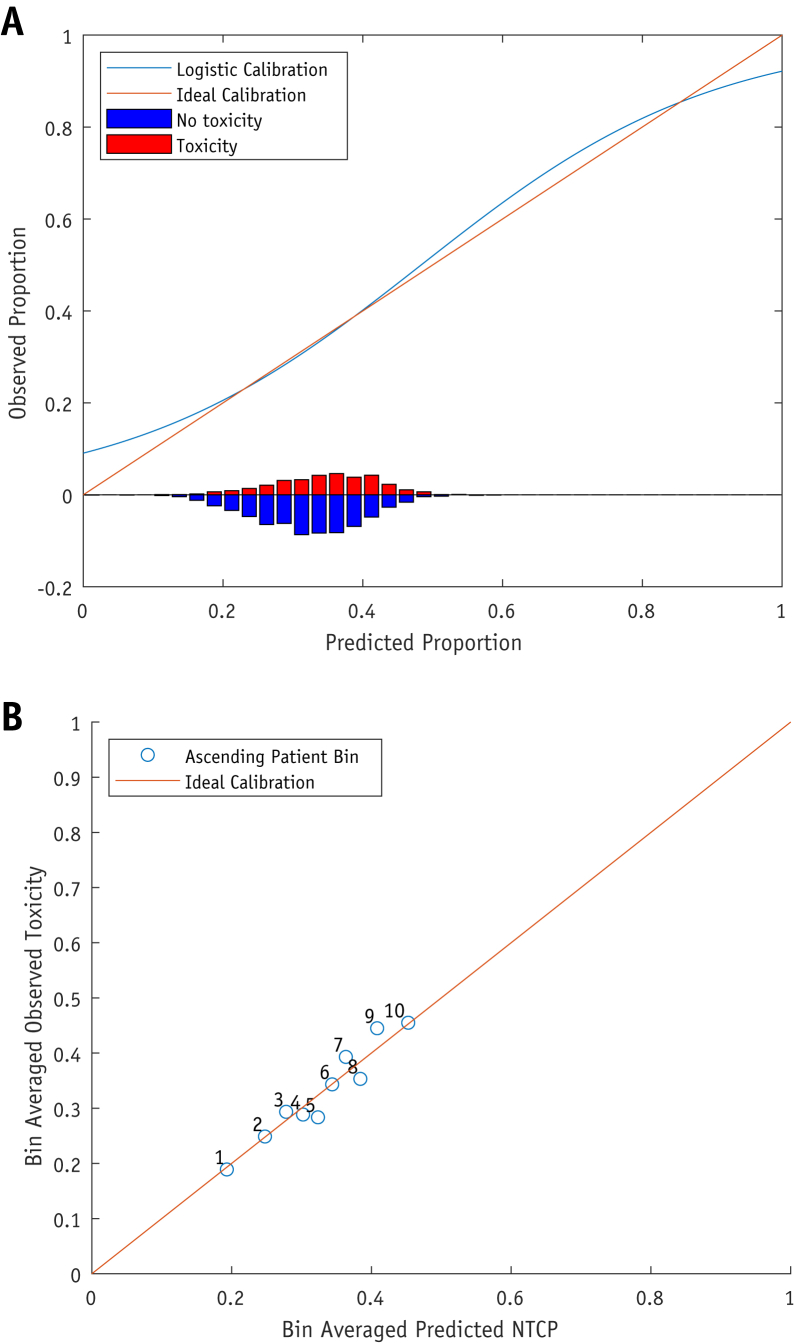
Calibration plots for rectal bleeding G1 + LKB-EQD2 model. (A) The fit of the model calibration (blue line) compared against optimal calibration (orange line), demonstrating a good overall fit. The lower histogram shows the predicted NTCP for patients, separated by toxicity (red, above line) or no toxicity (blue, below line). (B) Patients grouped into deciles by predicted NTCP, showing this against observed toxicity within each decile. Bin ordering is generally appropriate. (A color version of this figure is available at https://doi.org/10.1016/j.ijrobp.2020.12.041).

The calibration curve and binned calibration plot for the rectal bleeding G1+ LKB-EQD2 model is shown in Figure 2. Note that this is a well calibrated example. Calibration curves and binned calibration plots are presented for the LKB-EQD2 model fitted to each endpoint in Appendix E3 (Figs. E1-E16). The best calibrated models are those with the higher event rates (rectal bleeding G1+, stool frequency G1+, proctitis G1+). For those with lowest event rates (pain G1+, stricture/ulcer G1+), the calibration bin separation is less pronounced. Similar plots for the LKB-EQD2-DMF model, where it provided a statistically significant improvement in fit (IBD/diverticular disease for stool frequency G2+ and proctitis G1+) are presented in Appendix E4 (Figs. E17-E20). It can be seen that DMF inclusions cause higher decile risk bins to achieve better separation from other bins, compared with the equivalent LKB-EQD2 models without DMF (Figs. E6, E10).

One overall late rectal α/β ratio for use in the comparison of expected late rectal side effects between differing dose-fractionation schedules is desirable. The frequency weighted average for modelled late rectal G1+ events (excluding pain regarding poor fit) was α/β = 2.4 Gy and the equivalent for G2+ events was α/β = 2.3 Gy. Unfortunately, no transformation was found to normalize the highly positively skewed bootstrapped α/β ratio 95% CIs, meaning that pooling standard errors for a unified 95% CI is not appropriate.^39^ We would advise caution in the application of any single figure, since as demonstrated, the true fraction size sensitivity may differ between endpoints. The calculation of these estimates is shown in Table E3.

## Discussion

In this study, we have used data from a large phase 3 trial of moderately hypofractionated RT for nonmetastatic PCa. Through fitting an EQD2-corrected LKB model, estimates of the relative fraction size sensitivity (expressed as α/β ratio) for various clinician reported late rectal endpoints have been made. We have shown that these estimates do not vary markedly with inclusion of several possible dose modifying factors. To our knowledge, these are the first such individual rectal endpoint α/β ratio estimates in the literature.

Our α/β ratio estimates are generally lower than previous published articles with estimates of late rectal α/β ratio in humans. Brenner estimated late rectal RTOG G2+ α/β ratio = 5.4 Gy (95% CI, 3.9-6.9 Gy) using the proportions of patients experiencing toxicity from 8 dose-fractionation schedules in PCa EBRT studies in the United States and Japan.^18^ Dose heterogeneity was limited, with 2254 of 2306 patients receiving 1.8-2 Gy per fraction. Marzi et al^19^ used 162 patients from the Roma hypofractionation trial to model RTOG G2+ late rectal toxicity, estimating α/β = 2.3 Gy (95% CI, 1.1-5.6 Gy) using a similar LKB-EQD2 correction method to this study.^19^ However, fixed LKB parameters (*n* = 0.12; *m* = 0.15) were used during modeling, which artificially reduces CIs and might influence the α/β ratio estimate obtained. Tucker et al^20^ used 509 patients from RTOG 94 to 06, estimating late rectal RTOG G2+ α/β of 4.8 Gy, although with wide CIs (98% CI, 0.6-46 Gy).^20^ This wide estimate likely results from limited dose per fraction heterogeneity (1.8 and 2 Gy) and only 77 patients experiencing toxicity. In abstract form, Zhu et al^21^ reported data from 213 patients receiving conventional or moderately hypofractionated RT. Using an EQD2-corrected LKB model, they estimated G2+ LENT-SOM rectal α/β = 7.2 Gy (95% CI, 5.2-9.1), which is higher than other estimates.

Regarding the components of the traditional LKB model (*n*, *m*, *TD50*), it is reassuring that the LKB-NoEQD2 estimates for conventionally fractionated patients are similar to those previously reported for individual rectal endpoints.^7^^,^^40^, ^41^, ^42^ Estimates from these cohorts for bleeding, stool frequency, and proctitis are compared with our data in Table E4. The landmark QUANTEC study conducted a meta-analysis of LKB parameters from 4 of these studies, examining either G2 + rectal bleeding or G2 + late toxicity.^43^ Comparing our G2 + rectal bleeding LKB-NoEQD2 values for 74-Gy patients versus these QUANTEC meta-analysis values, we see fairly similar findings: n = 0.13 (95% CI, 0.01-0.42) versus 0.09 (95% CI, 0.04-0.14); m = 0.21 (95% CI, 0.06-0.43) versus 0.13 (95% CI, 0.10-0.17); and TD50 = 74.0 Gy (95% CI, 67.2-96.6) versus 76.9 Gy (95% CI, 73.7-80.1). Separately, we note that our models for pain produced wide CIs (eg, LKB-EQD2 α/β ratio estimate, 3.6 Gy; 95% CI, 0.01-840), suggestive of poor model fit for this endpoint. This is perhaps expected, given the relative subjectivity of pain.

Strengths of this study are drawn from the nature of the recorded data. The CHHiP trial is the largest study of hypofractionated RT for PCa, with two thirds of patients’ data being used for this analysis. We have included only patients reporting zero baseline toxicity, to reduce possible pre-existent toxicity noise. Furthermore, we have undertaken data quality assurance by checking every rectal contour for protocol adherence and recalculating DVHs. This large, clean sample, combined with multiple dose-fractionation regimens, has permitted α/β ratio estimation with tight CIs and good calibration for more frequently occurring endpoints—without the need to fix any of the parameters when modeling, as done previously.^19^ This study has also been aided by modern computing power facilitating the use of computationally intensive bootstrapping techniques. These techniques have facilitated nested model comparison using bootstrap-dependent estimates of test performance (632 estimate), reducing the potential influence of overfitting.

Limitations must also be considered, starting with the modeling approach itself. The LKB model is a traditional parametric method for the fitting of RT data, and more recent machine learning and artificial intelligence type modeling methodologies have been applied.^44^ The model does, however, facilitate fitting of data, with and without EQD2 correction, to estimate endpoint α/β ratios. Future toxicity modeling work with newer methodologies could benefit from these α/β ratio estimates, when using the linear-quadratic model to rescale DVH data predictors from disparate dose-fractionation regimens.

For the DMF coefficient estimates, it must be remembered that these have been estimated on cohorts in which those with baseline toxicity were excluded. Although the risk attributable to RT is hopefully more closely approximated, the absolute risk could be higher for those with a DMF for which disproportionately more patients were excluded for baseline toxicity (eg, hemorrhoids and rectal bleeding G1+; refer to Table E2).

An additional limitation is that motion has been demonstrated interfractionally for the rectum^45^ during prostate RT; therefore, the use of CT planned doses in this study is a limitation. We acknowledge that the endpoints modeled here are unlikely to recur in future trials, because of the amalgamation of multiple scales. This was a pragmatic choice based on the toxicity scales available, so there would be benefit to confirmatory studies with modern clinician reported scales (eg, Common Terminology Criteria for Adverse Events) or patient reported scales (eg, EPIC).^46^ Finally, despite the use of out-of-the-bag techniques, the data are from a single study, and future validation on another hypofractionated prostate RT data set would be desirable.

It is worth examining the α/β ratio assumptions (Table E5) and subsequent toxicity outcomes (Table E6) of the published phase 3 hypofractionation trials. The CHHiP Trial assumed a late rectal α/β ratio of 3 Gy and isoeffective design, with the 60- and 57-Gy arms reflecting uncertainty in the prostate α/β ratio (assumed α/β of 2.5 Gy and 1.5 Gy, respectively). The 60- and 57-Gy arms both showed nonsignificantly reduced cumulative rectal grade 2+ toxicity by 5 years (11.9% and 11.3% vs 13.7% for the control arm), with the 60-Gy arm being shown to be noninferior for disease control.^1^ PROFIT assumed late rectal α/β ratio = 3 to 5 Gy with isoeffective design (prostate α/β ratio, 1-3 Gy), achieving noninferior disease control with reduced late grade 2+ rectal toxicity in the test arm (8.9% vs 13.9%).^2^ RTOG 0415 assumed both tumor and late rectal α/β = 3 Gy, with the trial design escalating EQD2 to both.^3^ The trial achieved noninferior disease control with hypofractionation. Given the rectal dose escalation, the increased G2+ rectal toxicity in the hypofractionated arm (22.4% vs 14.0%) is not surprising. The HYPRO trial adopted an isotoxic design, assuming the highest α/β ratio for late rectal toxicity (α/β = 4-6 Gy). Unfortunately, this study demonstrated increased late G2+ rectal toxicity (21.9% vs 17.7%), without superior disease control. It is worth noting that HYPRO is the only phase 3 moderately hypofractionated study in which the relative test versus control late rectal toxicity was worse than anticipated in the trial design, most likely because of the higher assumed rectal α/β ratio and therefore dose delivered to the test arm.

Both large phase 3 randomized trials of prostate ultra-hypofractionation—PACE-B^47^ and HYPO-RT-PC^48^—have assumed a late rectal α/β of 3 Gy. The HYPO-RT-PC trial showed isoeffective cumulative grade 2 or worse late RTOG rectal toxicity for both arms: 42.7 Gy in 7 fractions (9.5%) and 78 Gy in 39 fractions (9.7%).^48^ The QUANTEC study on rectal toxicity also recommended dose adjustment by an α/β ratio of 3 Gy,^43^ an opinion that our data support. Corrected for multiple testing, our LKB-EQD2 models with freely fitted α/β ratios did not significantly outperform the same model with fixed α/β = 3 Gy. We do note that the upper bound of 95% CI for rectal bleeding G1+ was less than 3 Gy and that the results were close to corrected significance. This is perhaps worth noting, given that the randomized ProtecT trial showed bloody stools to be the most common patient-reported adverse event after RT compared with radical prostatectomy, although the long-term effects on bowel habits and bother were minimal.^49^

Future studies might use individual patient data–level analysis (accounting for baseline toxicity and dose distributions) of late toxicity from HYPO-RT-PC and, once released, PACE-B,^47^ to more definitively confirm applicability of the LQ model to late toxicity in ultra-hypofractionation, an area of some debate.^50^ It is possible that improving RT delivery techniques could lower rectal doses to less than the level at which fraction size sensitivity meaningfully influences toxicity.

## Conclusion

To our knowledge, this study is the first to provide α/β ratio estimates for individual late rectal toxicity endpoints seen after hypofractionated external beam RT for prostate cancer. Although symptom endpoints can occur concurrently, for G1+ rectal bleeding, one of the most objective endpoints, the α/β ratio 95% CI upper bound was <3 Gy. For G1+ endpoints, the frequency-weighted pooled estimate was late rectal α/β ratio = 2.4 Gy. However, adjusting for multiple testing, no significant improvement from an LKB-EQD2 model with α/β = 3 Gy was demonstrated. Future individual patient data level analysis on ultra-hypofractionated trials is desirable, but for now we suggest that a late rectal α/β ratio of no more than 3 Gy be used when comparing dose fractionation regimens.
