# Supplementary material for: Estimates of Alpha/Beta (α/β) Ratios for Individual Late Rectal Toxicity Endpoints: An Analysis of the CHHiP Trial
Source: Int J Radiat Oncol Biol Phys. 2021 Jun 1;110(2):596–608. doi: 10.1016/j.ijrobp.2020.12.041 (PMC8129972; doi:10.1016/j.ijrobp.2020.12.041)
Supplement: Supplementary Material [file mmc1.docx]

Supplementary Appendix

Contents

[Appendix A. Individual Late Rectal Endpoint Generation 3](#_Toc58525003)

[Table 1A. Constraints for Initial Grid Search in Fitted Models 8](#_Toc58525004)

[Appendix B. Rectal Cumulative DVHs by Dose-Fractionation Arm 11](#_Toc58525005)

[Table 2A. Proportions Expressing Categorical Dose-Modifying Factors Compared Between Patients Included, and Excluded Due to Baseline Toxicity 12](#_Toc58525006)

[Appendix C. Calibration Plots for LKB-EQD2 Model 13](#_Toc58525007)

[Figure 1A. Rectal Bleeding G2+ Calibration Curve: LKB-EQD2 Model 13](#_Toc58525008)

[Figure 2A. Rectal Bleeding G2+ Binned Calibration Plot: LKB-EQD2 Model 13](#_Toc58525009)

[Figure 3A. Stool Frequency G1+ Calibration Curve: LKB-EQD2 Model 14](#_Toc58525010)

[Figure 4A. Stool Frequency G1+ Binned Calibration Plot: LKB-EQD2 Model 14](#_Toc58525011)

[Figure 5A. Stool Frequency G2+ Calibration Curve: LKB-EQD2 Model 15](#_Toc58525012)

[Figure 6A. Stool Frequency G2+ Binned Calibration Plot: LKB-EQD2 Model 15](#_Toc58525013)

[Figure 7A. Pain G1+ Calibration Curve: LKB-EQD2 Model 16](#_Toc58525014)

[Figure 8A. Pain G1+ Binned Calibration Plot: LKB-EQD2 Model 16](#_Toc58525015)

[Figure 9A. Proctitis G1+ Calibration Curve: LKB-EQD2 Model 17](#_Toc58525016)

[Figure 10A. Proctitis G1+ Binned Calibration Plot: LKB-EQD2 Model 17](#_Toc58525017)

[Figure 11A. Proctitis G2+ Calibration Curve: LKB-EQD2 Model 18](#_Toc58525018)

[Figure 12A. Proctitis G2+ Binned Calibration Plot: LKB-EQD2 Model 18](#_Toc58525019)

[Figure 13A. Sphincter Control G1+ Calibration Curve: LKB-EQD2 Model 19](#_Toc58525020)

[Figure 14A. Sphincter Control G1+ Binned Calibration Plot: LKB-EQD2 Model 19](#_Toc58525021)

[Figure 15A. Stricture/Ulcer G1+ Calibration Curve: LKB-EQD2 Model 20](#_Toc58525022)

[Figure 16A. Stricture/Ulcer G1+ Binned Calibration Plot: LKB-EQD2 Model 20](#_Toc58525023)

[Appendix D. Calibration Plots for LKB-EQD2-DMF Models Significantly Improving on LKB-EQD2 Model 21](#_Toc58525024)

[Figure 17A. Stool Frequency G2+ Calibration Curve: LKB-EQD2-DMF Model (DMF = IBD/Diverticular) 21](#_Toc58525025)

[Figure 18A. Stool Frequency G2+ Binned Calibration Plot: LKB-EQD2-DMF Model (DMF = IBD/Diverticular) 21](#_Toc58525026)

[Figure 19A. Proctitis G1+ Calibration Curve: LKB-EQD2-DMF Model (DMF = IBD/Diverticular) 22](#_Toc58525027)

[Figure 20A. Proctitis G1+ Binned Calibration Plot: LKB-EQD2-DMF Model (DMF = IBD/Diverticular) 22](#_Toc58525028)

[Table 3A. Calculation of Pooled Rectal Late α/β Ratio 23](#_Toc58525029)

[Table 4A. LKB-NoEQD2 Parameter Comparison 24](#_Toc58525030)

[Table 5A. Moderate Hypofractionation Trial Design Assumptions 25](#_Toc58525031)

[Table 6A. Bowel Toxicity in Phase III Hypofractionation Trials 26](#_Toc58525032)

[Bibliography for Supplementary Appendix 27](#_Toc58525033)

# Appendix A. Individual Late Rectal Endpoint Generation

*Note on RTOG Scoring:*

In the trial follow-up forms, rather than an overall RTOG score, the possible contributory components were requested separately:

- Bowel obstruction
- Diarrhoea
- Proctitis
- Rectal-anal stricture
- Rectal ulcer

*Note on baseline scores*

The baseline score is generated as the WORST score of the baseline assessment and the pre-RT assessment. Patients would not be assigned a baseline score without the relevant endpoint being scored at one or both of those visits (and thus would be excluded from that endpoint). Only RMH and LENTSOM were collected at those timepoints, so RTOG scores are not considered in the adjudication of zero baseline toxicity.

*Endpoint generation*

The composite individual endpoints generated are listed, along with subdomain scores that would generate an event score in the composite endpoint. Exclusion criteria are explained.

**Bleeding G1+**

- Toxicity scored if:
  - Any ≥6 month f/u RMH Rectal bleeding (G1+) **OR**
  - Any ≥6 month f/u LENT-SOM Objective bleeding (G1+) **OR**
  - Any ≥6 month f/u LENT-SOM Management bleeding (G1+)
- Exclude unless:
  - Baseline RMH Rectal bleeding = G0 **AND**
  - Baseline LENT-SOM Objective bleeding = G0 **AND**
  - Baseline LENT-SOM Management bleeding = G0
- Exclude if missing >50% follow-up scores for any of:
  - RMH Rectal bleeding **OR**
  - LENT-SOM Objective bleeding **OR**
  - LENT-SOM Management bleeding

**Bleeding G2+**

- Toxicity scored if:
  - Any ≥6 month f/u RMH Rectal bleeding (G2+) **OR**
  - Any ≥6 month f/u LENT-SOM Objective bleeding (G2+) **OR**
  - Any ≥6 month f/u LENT-SOM Management bleeding (G1+)
- Exclude unless:
  - Baseline RMH Rectal bleeding = G0 **AND**
  - Baseline LENT-SOM Objective bleeding = G0 **AND**
  - Baseline LENT-SOM Management bleeding = G0
- Exclude if missing >50% (4/7) follow-up scores for any of:
  - RMH Rectal bleeding **OR**
  - LENT-SOM Objective bleeding **OR**
  - LENT-SOM Management bleeding

**Frequency G1+**

- Toxicity scored if:
  - Any ≥6 month f/u RTOG Diarrhoea (G1+) **OR**
  - Any ≥6 month f/u RMH Bowel frequency (G1+) **OR**
  - Any ≥6 month f/u LENT-SOM Subjective stool frequency (G1+) **OR**
  - Any ≥6 month f/u LENT-SOM Management tenesmus/stool freq. (G1+)
- Exclude unless:
  - Baseline RMH Bowel frequency = G0 **AND**
  - Baseline LENT-SOM Subjective stool frequency = G0 **AND**
  - Baseline LENT-SOM Management tenesmus/stool freq. = G0
- Exclude if missing >50% (4/7) follow-up scores for any of:
  - RTOG Diarrhoea **OR**
  - RMH Bowel frequency **OR**
  - LENT-SOM Subjective stool frequency **OR**
  - LENT-SOM Management tenesmus/stool freq.

**Frequency G2+**

- Toxicity scored if:
  - Any ≥6 month f/u RTOG Diarrhoea (G2+) **OR**
  - Any ≥6 month f/u RMH Bowel frequency (G2+) **OR**
  - Any ≥6 month f/u LENT-SOM Subjective stool frequency (G2+) **OR**
  - Any ≥6 month f/u LENT-SOM Management tenesmus/stool freq. (G1+)
- Exclude unless:
  - Baseline RMH Bowel frequency = G0 **AND**
  - Baseline LENT-SOM Subjective stool frequency = G0 **AND**
  - Baseline LENT-SOM Management tenesmus/stool freq. = G0
- Exclude if missing >50% (4/7) follow-up scores for any of:
  - RTOG Diarrhoea **OR**
  - RMH Bowel frequency **OR**
  - LENT-SOM Subjective stool frequency **OR**
  - LENT-SOM Management tenesmus/stool freq.

**Pain G1+**

- Toxicity scored if:
  - Any ≥6 month f/u LENT-SOM Subjective pain (G1+) **OR**
  - Any ≥6 month f/u LENT-SOM Management pain (G1+)
- Exclude unless:
  - Baseline LENT-SOM Subjective pain = G0 **AND**
  - Baseline LENT-SOM Management pain = G0
- Exclude if missing >50% (4/7) follow-up scores for any of:
  - LENT-SOM Subjective pain **OR**
  - LENT-SOM Management pain

**Proctitis G1+**

*Note: It was decided to include LENTSOM Management tenesmus / stool frequency in the stool frequency category. It could therefore not be included here to avoid double representation of that endpoint*

- Toxicity scored if:
  - Any ≥6 month f/u RTOG Proctitis (G1+) **OR**
  - Any ≥6 month f/u LENT-SOM Subjective tenesmus (G1+) **OR**
  - Any ≥6 month f/u LENT-SOM Subjective mucosal loss (G1+)
- Exclude unless:
  - Baseline LENT-SOM Subjective tenesmus = G0 **AND**
  - Baseline LENT-SOM Subjective mucosal loss = G0
- Exclude if missing >50% (4/7) follow-up scores for any of:
  - RTOG Proctitis **OR**
  - LENT-SOM Subjective tenesmus **OR**
  - LENT-SOM Subjective mucosal loss

**Proctitis G2+**

*Note: It was decided to include LENTSOM Management tenesmus / stool frequency in the stool frequency category. It could therefore not be included here to avoid double representation of that endpoint*

- Toxicity scored if:
  - Any ≥6 month f/u RTOG Proctitis (G2+) **OR**
  - Any ≥6 month f/u LENT-SOM Subjective tenesmus (G2+) **OR**
  - Any ≥6 month f/u LENT-SOM Subjective mucosal loss (G2+)
- Exclude unless:
  - Baseline LENT-SOM Subjective tenesmus = G0 **AND**
  - Baseline LENT-SOM Subjective mucosal loss = G0
- Exclude if missing >50% (4/7) follow-up scores for any of:
  - RTOG Proctitis **OR**
  - LENT-SOM Subjective tenesmus **OR**
  - LENT-SOM Subjective mucosal loss

**Sphincter Control G1+**

- Toxicity scored if:
  - Any ≥6 month f/u LENT-SOM Subjective sphincter control (G1+) **OR**
  - Any ≥6 month f/u LENT-SOM Management sphincter control (G1+)
- Exclude unless:
  - Baseline LENT-SOM Subjective sphincter control = G0 **AND**
  - Baseline LENT-SOM Management sphincter control = G0
- Exclude if missing >50% (4/7) follow-up scores for any of:
  - LENT-SOM Subjective sphincter control **OR**
  - LENT-SOM Management sphincter control

**Stricture/Ulcer G1+**

- Toxicity scored if:
  - Any ≥6 month f/u RTOG bowel obstruction (G1+) **OR**
  - Any ≥6 month f/u RTOG rectal-anal stricture (G1+) **OR**
  - Any ≥6 month f/u RTOG rectal ulcer (G1+) **OR**
  - Any ≥6 month f/u LENT-SOM Objective ulceration (G1+) **OR**
  - Any ≥6 month f/u LENT-SOM Objective stricture (G1+) **OR**
  - Any ≥6 month f/u LENT-SOM Management ulceration (G1+) **OR**
  - Any ≥6 month f/u LENT-SOM Management stricture (G1+)
- Exclude unless:
  - Baseline LENT-SOM Objective ulceration = G0 **AND**
  - Baseline LENT-SOM Objective stricture = G0 **AND**
  - Baseline LENT-SOM Management ulceration = G0 **AND**
  - Baseline LENT-SOM Management stricture = G0
- Exclude if missing >50% (4/7) follow-up scores for any of:
  - RTOG bowel obstruction **OR**
  - RTOG rectal-anal stricture **OR**
  - RTOG rectal ulcer **OR**
  - LENT-SOM Objective ulceration **OR**
  - LENT-SOM Objective stricture **OR**
  - LENT-SOM Management ulceration **OR**
  - LENT-SOM Management stricture

# Table 1A. Constraints for Initial Grid Search in Fitted Models

| **Endpoint Name** | **Patients** | **Model Type** | **DMF** | **n**  **LB** | **n**  **UB** | **n step** | **m**  **LB** | **m**  **UB** | **m**  **step** | **TD50**  **LB** | **TD50**  **UB** | **TD50**  **step** | **α/β**  **LB** | **α/β**  **UB** | **α/β**  **step** | **DMF**  **LB** | **DMF**  **UB** | **DMF**  **step** |
| --- | --- | --- | --- | --- | --- | --- | --- | --- | --- | --- | --- | --- | --- | --- | --- | --- | --- | --- |
| **Rectal Bleeding G1+** | 57 & 60 | No EQD2 | nil | 0.05 | 0.95 | 0.05 | 0.05 | 0.95 | 0.05 | 30 | 90 | 3 | N/A | N/A | N/A | N/A | N/A | N/A |
| **Rectal Bleeding G1+** | 74 | No EQD2 | nil | 0.05 | 0.95 | 0.05 | 0.05 | 3 | 0.05 | 30 | 90 | 3 | N/A | N/A | N/A | N/A | N/A | N/A |
| **Rectal Bleeding G1+** | All | EQD2 | nil | 0.05 | 0.95 | 0.05 | 0.05 | 0.95 | 0.05 | 30 | 90 | 3 | 0.2 | 6.2 | 0.4 | N/A | N/A | N/A |
| **Rectal Bleeding G1+** | All | EQD2 | Age | 0.05 | 0.95 | 0.05 | 0.05 | 0.95 | 0.05 | 30 | 90 | 3 | 0.2 | 6.2 | 0.4 | -0.018 | 0.009 | 0.003 |
| **Rectal Bleeding G1+** | All | EQD2 | Diabetes | 0.05 | 0.95 | 0.05 | 0.05 | 0.95 | 0.05 | 30 | 90 | 3 | 0.2 | 4.2 | 0.4 | -0.28 | 0.12 | 0.04 |
| **Rectal Bleeding G1+** | All | EQD2 | Hypertension | 0.05 | 0.95 | 0.05 | 0.05 | 0.95 | 0.05 | 30 | 90 | 3 | 0.2 | 5 | 0.4 | -0.15 | 0.15 | 0.03 |
| **Rectal Bleeding G1+** | All | EQD2 | IBD/Diverticular | 0.05 | 0.95 | 0.05 | 0.05 | 0.85 | 0.05 | 30 | 90 | 3 | 0.2 | 4.2 | 0.4 | -0.12 | 0.45 | 0.03 |
| **Rectal Bleeding G1+** | All | EQD2 | Pelvic Surgery | 0.05 | 0.95 | 0.05 | 0.05 | 0.85 | 0.05 | 30 | 90 | 3 | 0.2 | 5 | 0.4 | -0.12 | 0.33 | 0.03 |
| **Rectal Bleeding G1+** | All | EQD2 | Haemorrhoids | 0.05 | 0.95 | 0.05 | 0.05 | 0.95 | 0.05 | 30 | 90 | 3 | 0.2 | 5 | 0.4 | -0.24 | 0.36 | 0.04 |
| **Rectal Bleeding G2+** | 57 & 60 | No EQD2 | nil | 0.05 | 0.95 | 0.05 | 0.05 | 0.95 | 0.05 | 30 | 90 | 3 | N/A | N/A | N/A | N/A | N/A | N/A |
| **Rectal Bleeding G2+** | 74 | No EQD2 | nil | 0.05 | 0.95 | 0.05 | 0.05 | 3 | 0.05 | 30 | 90 | 3 | N/A | N/A | N/A | N/A | N/A | N/A |
| **Rectal Bleeding G2+** | All | EQD2 | nil | 0.05 | 0.95 | 0.05 | 0.05 | 0.95 | 0.05 | 30 | 90 | 3 | 0.2 | 6.2 | 0.4 | N/A | N/A | N/A |
| **Rectal Bleeding G2+** | All | EQD2 | Age | 0.05 | 0.95 | 0.05 | 0.05 | 0.95 | 0.05 | 30 | 90 | 3 | 0.2 | 6.2 | 0.4 | -0.018 | 0.009 | 0.003 |
| **Rectal Bleeding G2+** | All | EQD2 | Diabetes | 0.05 | 0.95 | 0.05 | 0.05 | 0.95 | 0.05 | 30 | 90 | 3 | 0.2 | 4.2 | 0.4 | -0.28 | 0.12 | 0.04 |
| **Rectal Bleeding G2+** | All | EQD2 | Hypertension | 0.05 | 0.95 | 0.05 | 0.05 | 0.95 | 0.05 | 30 | 90 | 3 | 0.2 | 5 | 0.4 | -0.15 | 0.15 | 0.03 |
| **Rectal Bleeding G2+** | All | EQD2 | IBD/Diverticular | 0.05 | 0.95 | 0.05 | 0.05 | 0.85 | 0.05 | 30 | 90 | 3 | 0.2 | 4.2 | 0.4 | -0.12 | 0.45 | 0.03 |
| **Rectal Bleeding G2+** | All | EQD2 | Pelvic Surgery | 0.05 | 0.95 | 0.05 | 0.05 | 0.85 | 0.05 | 30 | 90 | 3 | 0.2 | 5 | 0.4 | -0.12 | 0.33 | 0.03 |
| **Rectal Bleeding G2+** | All | EQD2 | Haemorrhoids | 0.05 | 0.95 | 0.05 | 0.05 | 0.95 | 0.05 | 30 | 90 | 3 | 0.2 | 5 | 0.4 | -0.24 | 0.36 | 0.04 |
| **Pain G1+** | 57 & 60 | No EQD2 | nil | 0.05 | 0.95 | 0.05 | 0.05 | 0.95 | 0.05 | 30 | 90 | 3 | N/A | N/A | N/A | N/A | N/A | N/A |
| **Pain G1+** | 74 | No EQD2 | nil | 0.05 | 0.95 | 0.05 | 0.05 | 3 | 0.05 | 30 | 90 | 3 | N/A | N/A | N/A | N/A | N/A | N/A |
| **Pain G1+** | All | EQD2 | nil | 0.05 | 0.95 | 0.05 | 0.05 | 0.95 | 0.05 | 30 | 90 | 3 | 0.2 | 6.2 | 0.4 | N/A | N/A | N/A |
| **Pain G1+** | All | EQD2 | Age | 0.05 | 0.95 | 0.05 | 0.05 | 0.95 | 0.05 | 30 | 90 | 3 | 0.2 | 6.2 | 0.4 | -0.018 | 0.009 | 0.003 |
| **Pain G1+** | All | EQD2 | Diabetes | 0.05 | 0.95 | 0.05 | 0.05 | 0.95 | 0.05 | 30 | 90 | 3 | 0.2 | 4.2 | 0.4 | -0.28 | 0.12 | 0.04 |
| **Pain G1+** | All | EQD2 | Hypertension | 0.05 | 0.95 | 0.05 | 0.05 | 0.95 | 0.05 | 30 | 90 | 3 | 0.2 | 5 | 0.4 | -0.15 | 0.15 | 0.03 |
| **Pain G1+** | All | EQD2 | IBD/Diverticular | 0.05 | 0.95 | 0.05 | 0.05 | 0.85 | 0.05 | 30 | 90 | 3 | 0.2 | 4.2 | 0.4 | -0.12 | 0.45 | 0.03 |
| **Pain G1+** | All | EQD2 | Pelvic Surgery | 0.05 | 0.95 | 0.05 | 0.05 | 0.85 | 0.05 | 30 | 90 | 3 | 0.2 | 5 | 0.4 | -0.12 | 0.33 | 0.03 |
| **Pain G1+** | All | EQD2 | Haemorrhoids | 0.05 | 0.95 | 0.05 | 0.05 | 0.95 | 0.05 | 30 | 90 | 3 | 0.2 | 5 | 0.4 | -0.24 | 0.36 | 0.04 |
| **Table 1A continued…** | | | | | | | | | | | | | | | | | | |
| **Endpoint Name** | **Patients** | **Model Type** | **DMF** | **n**  **LB** | **n**  **UB** | **n step** | **m**  **LB** | **m**  **UB** | **m**  **step** | **TD50**  **LB** | **TD50**  **UB** | **TD50**  **step** | **α/β**  **LB** | **α/β**  **UB** | **α/β**  **step** | **DMF**  **LB** | **DMF**  **UB** | **DMF**  **step** |
| **Proctitis G1+** | 57 & 60 | No EQD2 | nil | 0.05 | 0.95 | 0.05 | 0.05 | 0.95 | 0.05 | 30 | 90 | 3 | N/A | N/A | N/A | N/A | N/A | N/A |
| **Proctitis G1+** | 74 | No EQD2 | nil | 0.05 | 0.95 | 0.05 | 0.05 | 3 | 0.05 | 30 | 90 | 3 | N/A | N/A | N/A | N/A | N/A | N/A |
| **Proctitis G1+** | All | EQD2 | nil | 0.05 | 0.95 | 0.05 | 0.05 | 0.95 | 0.05 | 30 | 90 | 3 | 0.2 | 6.2 | 0.4 | N/A | N/A | N/A |
| **Proctitis G1+** | All | EQD2 | Age | 0.05 | 0.95 | 0.05 | 0.05 | 0.95 | 0.05 | 30 | 90 | 3 | 0.2 | 6.2 | 0.4 | -0.018 | 0.009 | 0.003 |
| **Proctitis G1+** | All | EQD2 | Diabetes | 0.05 | 0.95 | 0.05 | 0.05 | 0.95 | 0.05 | 30 | 90 | 3 | 0.2 | 4.2 | 0.4 | -0.28 | 0.12 | 0.04 |
| **Proctitis G1+** | All | EQD2 | Hypertension | 0.05 | 0.95 | 0.05 | 0.05 | 0.95 | 0.05 | 30 | 90 | 3 | 0.2 | 5 | 0.4 | -0.15 | 0.15 | 0.03 |
| **Proctitis G1+** | All | EQD2 | IBD/Diverticular | 0.05 | 0.95 | 0.05 | 0.05 | 0.85 | 0.05 | 30 | 90 | 3 | 0.2 | 4.2 | 0.4 | -0.12 | 0.45 | 0.03 |
| **Proctitis G1+** | All | EQD2 | Pelvic Surgery | 0.05 | 0.95 | 0.05 | 0.05 | 0.85 | 0.05 | 30 | 90 | 3 | 0.2 | 5 | 0.4 | -0.12 | 0.33 | 0.03 |
| **Proctitis G1+** | All | EQD2 | Haemorrhoids | 0.05 | 0.95 | 0.05 | 0.05 | 0.95 | 0.05 | 30 | 90 | 3 | 0.2 | 5 | 0.4 | -0.24 | 0.36 | 0.04 |
| **Proctitis G2+** | 57 & 60 | No EQD2 | nil | 0.05 | 0.95 | 0.05 | 0.05 | 0.95 | 0.05 | 30 | 90 | 3 | N/A | N/A | N/A | N/A | N/A | N/A |
| **Proctitis G2+** | 74 | No EQD2 | nil | 0.05 | 0.95 | 0.05 | 0.05 | 3 | 0.05 | 30 | 90 | 3 | N/A | N/A | N/A | N/A | N/A | N/A |
| **Proctitis G2+** | All | EQD2 | nil | 0.05 | 0.95 | 0.05 | 0.05 | 0.95 | 0.05 | 30 | 90 | 3 | 0.2 | 6.2 | 0.4 | N/A | N/A | N/A |
| **Proctitis G2+** | All | EQD2 | Age | 0.05 | 0.95 | 0.05 | 0.05 | 0.95 | 0.05 | 30 | 90 | 3 | 0.2 | 6.2 | 0.4 | -0.018 | 0.009 | 0.003 |
| **Proctitis G2+** | All | EQD2 | Diabetes | 0.05 | 0.95 | 0.05 | 0.05 | 0.95 | 0.05 | 30 | 90 | 3 | 0.2 | 4.2 | 0.4 | -0.28 | 0.12 | 0.04 |
| **Proctitis G2+** | All | EQD2 | Hypertension | 0.05 | 0.95 | 0.05 | 0.05 | 0.95 | 0.05 | 30 | 90 | 3 | 0.2 | 5 | 0.4 | -0.15 | 0.15 | 0.03 |
| **Proctitis G2+** | All | EQD2 | IBD/Diverticular | 0.05 | 0.95 | 0.05 | 0.05 | 0.85 | 0.05 | 30 | 90 | 3 | 0.2 | 4.2 | 0.4 | -0.12 | 0.45 | 0.03 |
| **Proctitis G2+** | All | EQD2 | Pelvic Surgery | 0.05 | 0.95 | 0.05 | 0.05 | 0.85 | 0.05 | 30 | 90 | 3 | 0.2 | 5 | 0.4 | -0.12 | 0.33 | 0.03 |
| **Proctitis G2+** | All | EQD2 | Haemorrhoids | 0.05 | 0.95 | 0.05 | 0.05 | 0.95 | 0.05 | 30 | 90 | 3 | 0.2 | 5 | 0.4 | -0.24 | 0.36 | 0.04 |
| **Sphincter Control G1+** | 57 & 60 | No EQD2 | nil | 0.05 | 0.95 | 0.05 | 0.05 | 0.95 | 0.05 | 30 | 90 | 3 | N/A | N/A | N/A | N/A | N/A | N/A |
| **Sphincter Control G1+** | 74 | No EQD2 | nil | 0.05 | 0.95 | 0.05 | 0.05 | 3 | 0.05 | 30 | 90 | 3 | N/A | N/A | N/A | N/A | N/A | N/A |
| **Sphincter Control G1+** | All | EQD2 | nil | 0.05 | 0.95 | 0.05 | 0.05 | 0.95 | 0.05 | 30 | 90 | 3 | 0.2 | 6.2 | 0.4 | N/A | N/A | N/A |
| **Sphincter Control G1+** | All | EQD2 | Age | 0.05 | 0.95 | 0.05 | 0.05 | 0.95 | 0.05 | 30 | 90 | 3 | 0.2 | 6.2 | 0.4 | -0.018 | 0.009 | 0.003 |
| **Sphincter Control G1+** | All | EQD2 | Diabetes | 0.05 | 0.95 | 0.05 | 0.05 | 0.95 | 0.05 | 30 | 90 | 3 | 0.2 | 4.2 | 0.4 | -0.28 | 0.12 | 0.04 |
| **Sphincter Control G1+** | All | EQD2 | Hypertension | 0.05 | 0.95 | 0.05 | 0.05 | 0.95 | 0.05 | 30 | 90 | 3 | 0.2 | 5 | 0.4 | -0.15 | 0.15 | 0.03 |
| **Sphincter Control G1+** | All | EQD2 | IBD/Diverticular | 0.05 | 0.95 | 0.05 | 0.05 | 0.85 | 0.05 | 30 | 90 | 3 | 0.2 | 4.2 | 0.4 | -0.12 | 0.45 | 0.03 |
| **Sphincter Control G1+** | All | EQD2 | Pelvic Surgery | 0.05 | 0.95 | 0.05 | 0.05 | 0.85 | 0.05 | 30 | 90 | 3 | 0.2 | 5 | 0.4 | -0.12 | 0.33 | 0.03 |
| **Sphincter Control G1+** | All | EQD2 | Haemorrhoids | 0.05 | 0.95 | 0.05 | 0.05 | 0.95 | 0.05 | 30 | 90 | 3 | 0.2 | 5 | 0.4 | -0.24 | 0.36 | 0.04 |
| **Table 1A continued…** | | | | | | | | | | | | | | | | | | |
| **Endpoint Name** | Patients | Model Type | DMF | n  LB | n  UB | n step | m  LB | m  UB | m  step | TD50  LB | TD50  UB | TD50  step | α/β  LB | α/β  UB | α/β  step | DMF  LB | DMF  UB | DMF  step |
| **Stool Frequency G1+** | 57 & 60 | No EQD2 | nil | 0.05 | 0.95 | 0.05 | 0.05 | 0.95 | 0.05 | 30 | 90 | 3 | N/A | N/A | N/A | N/A | N/A | N/A |
| **Stool Frequency G1+** | 74 | No EQD2 | nil | 0.05 | 0.95 | 0.05 | 0.05 | 3 | 0.05 | 30 | 90 | 3 | N/A | N/A | N/A | N/A | N/A | N/A |
| **Stool Frequency G1+** | All | EQD2 | nil | 0.05 | 0.95 | 0.05 | 0.05 | 0.95 | 0.05 | 30 | 90 | 3 | 0.2 | 6.2 | 0.4 | N/A | N/A | N/A |
| **Stool Frequency G1+** | All | EQD2 | Age | 0.05 | 0.95 | 0.05 | 0.05 | 0.95 | 0.05 | 30 | 90 | 3 | 0.2 | 6.2 | 0.4 | -0.018 | 0.009 | 0.003 |
| **Stool Frequency G1+** | All | EQD2 | Diabetes | 0.05 | 0.95 | 0.05 | 0.05 | 0.95 | 0.05 | 30 | 90 | 3 | 0.2 | 4.2 | 0.4 | -0.28 | 0.12 | 0.04 |
| **Stool Frequency G1+** | All | EQD2 | Hypertension | 0.05 | 0.95 | 0.05 | 0.05 | 0.95 | 0.05 | 30 | 90 | 3 | 0.2 | 5 | 0.4 | -0.15 | 0.15 | 0.03 |
| **Stool Frequency G1+** | All | EQD2 | IBD/Diverticular | 0.05 | 0.95 | 0.05 | 0.05 | 0.85 | 0.05 | 30 | 90 | 3 | 0.2 | 4.2 | 0.4 | -0.12 | 0.45 | 0.03 |
| **Stool Frequency G1+** | All | EQD2 | Pelvic Surgery | 0.05 | 0.95 | 0.05 | 0.05 | 0.85 | 0.05 | 30 | 90 | 3 | 0.2 | 5 | 0.4 | -0.12 | 0.33 | 0.03 |
| **Stool Frequency G1+** | All | EQD2 | Haemorrhoids | 0.05 | 0.95 | 0.05 | 0.05 | 0.95 | 0.05 | 30 | 90 | 3 | 0.2 | 5 | 0.4 | -0.24 | 0.36 | 0.04 |
| **Stool Frequency G2+** | 57 & 60 | No EQD2 | nil | 0.05 | 0.95 | 0.05 | 0.05 | 0.95 | 0.05 | 30 | 90 | 3 | N/A | N/A | N/A | N/A | N/A | N/A |
| **Stool Frequency G2+** | 74 | No EQD2 | nil | 0.05 | 0.95 | 0.05 | 0.05 | 3 | 0.05 | 30 | 90 | 3 | N/A | N/A | N/A | N/A | N/A | N/A |
| **Stool Frequency G2+** | All | EQD2 | nil | 0.05 | 0.95 | 0.05 | 0.05 | 0.95 | 0.05 | 30 | 90 | 3 | 0.2 | 6.2 | 0.4 | N/A | N/A | N/A |
| **Stool Frequency G2+** | All | EQD2 | Age | 0.05 | 0.95 | 0.05 | 0.05 | 0.95 | 0.05 | 30 | 90 | 3 | 0.2 | 6.2 | 0.4 | -0.018 | 0.009 | 0.003 |
| **Stool Frequency G2+** | All | EQD2 | Diabetes | 0.05 | 0.95 | 0.05 | 0.05 | 0.95 | 0.05 | 30 | 90 | 3 | 0.2 | 4.2 | 0.4 | -0.28 | 0.12 | 0.04 |
| **Stool Frequency G2+** | All | EQD2 | Hypertension | 0.05 | 0.95 | 0.05 | 0.05 | 0.95 | 0.05 | 30 | 90 | 3 | 0.2 | 5 | 0.4 | -0.15 | 0.15 | 0.03 |
| **Stool Frequency G2+** | All | EQD2 | IBD/Diverticular | 0.05 | 0.95 | 0.05 | 0.05 | 0.85 | 0.05 | 30 | 90 | 3 | 0.2 | 4.2 | 0.4 | -0.12 | 0.45 | 0.03 |
| **Stool Frequency G2+** | All | EQD2 | Pelvic Surgery | 0.05 | 0.95 | 0.05 | 0.05 | 0.85 | 0.05 | 30 | 90 | 3 | 0.2 | 5 | 0.4 | -0.12 | 0.33 | 0.03 |
| **Stool Frequency G2+** | All | EQD2 | Haemorrhoids | 0.05 | 0.95 | 0.05 | 0.05 | 0.95 | 0.05 | 30 | 90 | 3 | 0.2 | 5 | 0.4 | -0.24 | 0.36 | 0.04 |
| **Stricture/Ulcer G1+** | 57 & 60 | No EQD2 | nil | 0.05 | 0.95 | 0.05 | 0.05 | 0.95 | 0.05 | 30 | 90 | 3 | N/A | N/A | N/A | N/A | N/A | N/A |
| **Stricture/Ulcer G1+** | 74 | No EQD2 | nil | 0.05 | 0.95 | 0.05 | 0.05 | 3 | 0.05 | 30 | 90 | 3 | N/A | N/A | N/A | N/A | N/A | N/A |
| **Stricture/Ulcer G1+** | All | EQD2 | nil | 0.05 | 0.95 | 0.05 | 0.05 | 0.95 | 0.05 | 30 | 90 | 3 | 0.2 | 6.2 | 0.4 | N/A | N/A | N/A |
| **Stricture/Ulcer G1+** | All | EQD2 | Age | 0.05 | 0.95 | 0.05 | 0.05 | 0.95 | 0.05 | 30 | 90 | 3 | 0.2 | 6.2 | 0.4 | -0.018 | 0.009 | 0.003 |
| **Stricture/Ulcer G1+** | All | EQD2 | Diabetes | 0.05 | 0.95 | 0.05 | 0.05 | 0.95 | 0.05 | 30 | 90 | 3 | 0.2 | 4.2 | 0.4 | -0.28 | 0.12 | 0.04 |
| **Stricture/Ulcer G1+** | All | EQD2 | Hypertension | 0.05 | 0.95 | 0.05 | 0.05 | 0.95 | 0.05 | 30 | 90 | 3 | 0.2 | 5 | 0.4 | -0.15 | 0.15 | 0.03 |
| **Stricture/Ulcer G1+** | All | EQD2 | IBD/Diverticular | 0.05 | 0.95 | 0.05 | 0.05 | 0.85 | 0.05 | 30 | 90 | 3 | 0.2 | 4.2 | 0.4 | -0.12 | 0.45 | 0.03 |
| **Stricture/Ulcer G1+** | All | EQD2 | Pelvic Surgery | 0.05 | 0.95 | 0.05 | 0.05 | 0.85 | 0.05 | 30 | 90 | 3 | 0.2 | 5 | 0.4 | -0.12 | 0.33 | 0.03 |
| **Stricture/Ulcer G1+** | All | EQD2 | Haemorrhoids | 0.05 | 0.95 | 0.05 | 0.05 | 0.95 | 0.05 | 30 | 90 | 3 | 0.2 | 5 | 0.4 | -0.24 | 0.36 | 0.04 |

# Appendix B. Rectal Cumulative DVHs by Dose-Fractionation Arm


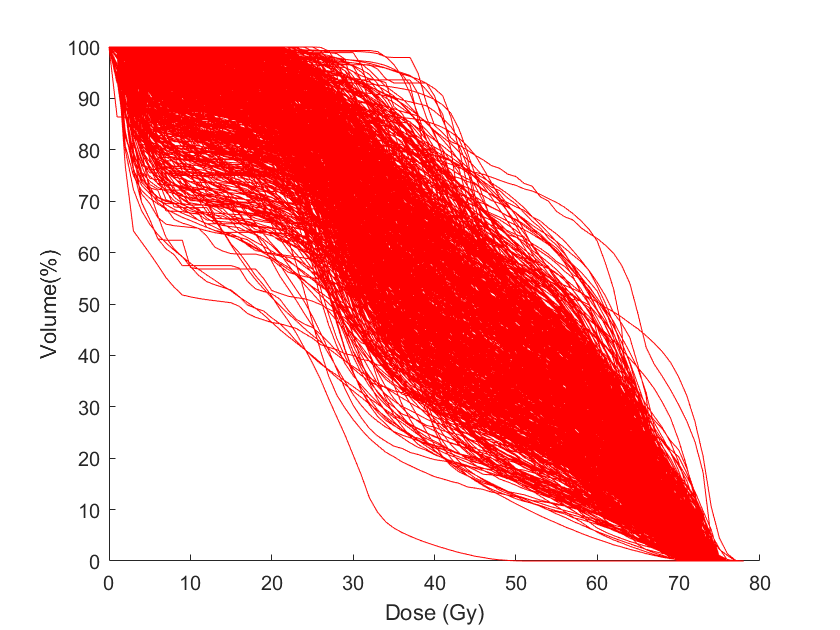


*

74 Gy in 37 Fraction Patients

n=711

*Single very low DVH due to good patient anatomy and excellent Tomotherapy plan.


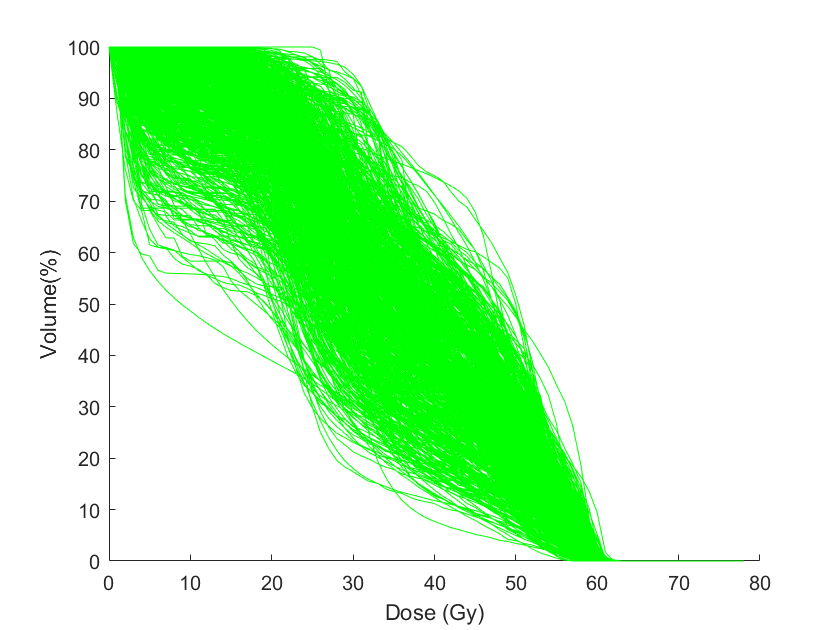


60 Gy in 20 Fraction Patients

n=752


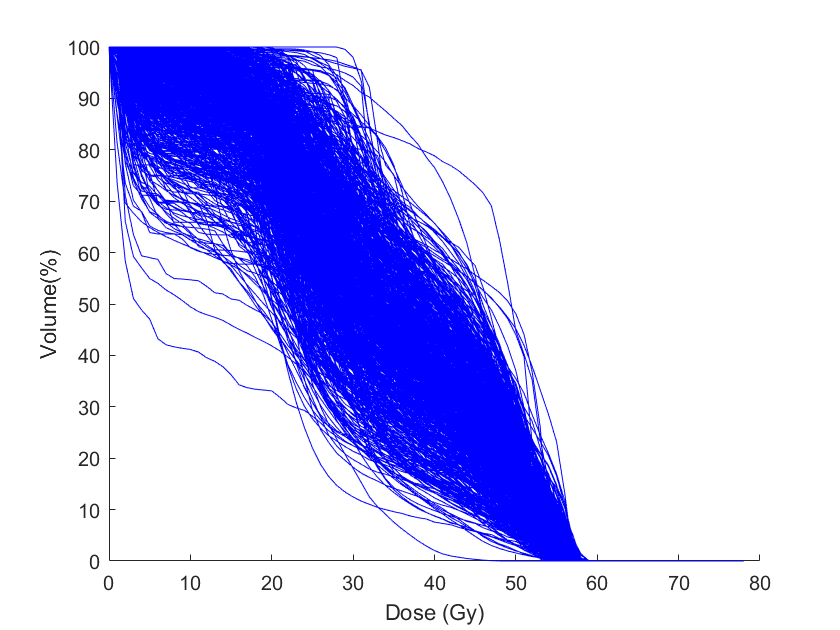


57 Gy in 19 Fraction Patients

n=752

# Table 2A. Proportions Expressing Categorical Dose-Modifying Factors Compared Between Patients Included, and Excluded Due to Baseline Toxicity

| Categorical Dose Modifying Factor | Endpoint | Included  (n) | DMF presence amongst included  (%) | Excluded for baseline toxicity  (n) | DMF presence amongst excluded  (%) | Chi-square p-val |
| --- | --- | --- | --- | --- | --- | --- |
| Diabetes | Bleeding G1+ | 2008 | 10.4% | 206 | 8.7% | 0.4643 |
|  | Bleeding G2+ | 2006 | 10.4% | 206 | 8.7% | 0.4617 |
|  | Frequency G1+ | 2025 | 10.4% | 176 | 10.8% | 0.8594 |
|  | Frequency G2+ | 2021 | 10.3% | 176 | 10.8% | 0.8498 |
|  | Pain G1+ | 2185 | 10.3% | 28 | 3.6% | 0.2406 |
|  | Proctitis G1+ | 2147 | 10.4% | 70 | 7.1% | 0.3793 |
|  | Proctitis G2+ | 2146 | 10.4% | 70 | 7.1% | 0.3787 |
|  | Sphincter Control G1+ | 2199 | 10.2% | 14 | 21.4% | 0.1695 |
|  | Stricture/Ulcer G1+ | 2206 | 10.2% | 2 | 0.0% | 0.6328 |
| Hypertension | Bleeding G1+ | 2008 | 39.2% | 206 | 41.3% | 0.5723 |
|  | Bleeding G2+ | 2006 | 39.2% | 206 | 41.3% | 0.5702 |
|  | Frequency G1+ | 2025 | 39.5% | 176 | 39.8% | 0.9447 |
|  | Frequency G2+ | 2021 | 39.4% | 176 | 39.8% | 0.9301 |
|  | Pain G1+ | 2185 | 39.6% | 28 | 28.6% | 0.2341 |
|  | Proctitis G1+ | 2147 | 39.2% | 70 | 45.7% | 0.2737 |
|  | Proctitis G2+ | 2146 | 39.2% | 70 | 45.7% | 0.2751 |
|  | Sphincter Control G1+ | 2199 | 39.4% | 14 | 50.0% | 0.4177 |
|  | Stricture/Ulcer G1+ | 2206 | 39.4% | 2 | 50.0% | 0.7600 |
| IBD or Diverticular Disease | Bleeding G1+ | 2008 | 3.3% | 206 | 8.7% | **0.0001** |
|  | Bleeding G2+ | 2006 | 3.3% | 206 | 8.7% | **0.0001** |
|  | Frequency G1+ | 2025 | 3.5% | 176 | 6.3% | 0.0652 |
|  | Frequency G2+ | 2021 | 3.5% | 176 | 6.3% | 0.0662 |
|  | Pain G1+ | 2185 | 3.8% | 28 | 7.1% | 0.3602 |
|  | Proctitis G1+ | 2147 | 3.7% | 70 | 7.1% | 0.1429 |
|  | Proctitis G2+ | 2146 | 3.7% | 70 | 7.1% | 0.1432 |
|  | Sphincter Control G1+ | 2199 | 3.8% | 14 | 7.1% | 0.5190 |
|  | Stricture/Ulcer G1+ | 2206 | 3.9% | 2 | 0.0% | 0.7771 |
| Pelvic Surgery | Bleeding G1+ | 2008 | 7.3% | 206 | 9.7% | 0.2058 |
|  | Bleeding G2+ | 2006 | 7.3% | 206 | 9.7% | 0.2073 |
|  | Frequency G1+ | 2025 | 7.2% | 176 | 10.2% | 0.1362 |
|  | Frequency G2+ | 2021 | 7.2% | 176 | 10.2% | 0.1383 |
|  | Pain G1+ | 2185 | 7.3% | 28 | 10.7% | 0.4948 |
|  | Proctitis G1+ | 2147 | 7.2% | 70 | 12.9% | 0.0761 |
|  | Proctitis G2+ | 2146 | 7.2% | 70 | 12.9% | 0.0764 |
|  | Sphincter Control G1+ | 2199 | 7.4% | 14 | 7.1% | 0.9745 |
|  | Stricture/Ulcer G1+ | 2206 | 7.3% | 2 | 50.0% | **0.0211** |
| Haemorrhoids | Bleeding G1+ | 2008 | 4.2% | 206 | 35.0% | **0.0000** |
|  | Bleeding G2+ | 2006 | 4.2% | 206 | 35.0% | **0.0000** |
|  | Frequency G1+ | 2025 | 6.6% | 176 | 10.8% | **0.0339** |
|  | Frequency G2+ | 2021 | 6.5% | 176 | 10.8% | **0.0320** |
|  | Pain G1+ | 2185 | 6.7% | 28 | 25.0% | **0.0001** |
|  | Proctitis G1+ | 2147 | 6.8% | 70 | 12.9% | **0.0481** |
|  | Proctitis G2+ | 2146 | 6.8% | 70 | 12.9% | **0.0483** |
|  | Sphincter Control G1+ | 2199 | 6.9% | 14 | 7.1% | 0.9730 |
|  | Stricture/Ulcer G1+ | 2206 | 6.9% | 2 | 0.0% | 0.6995 |

# Appendix C. Calibration Plots for LKB-EQD2 Model

## Figure 1A. Rectal Bleeding G2+ Calibration Curve: LKB-EQD2 Model


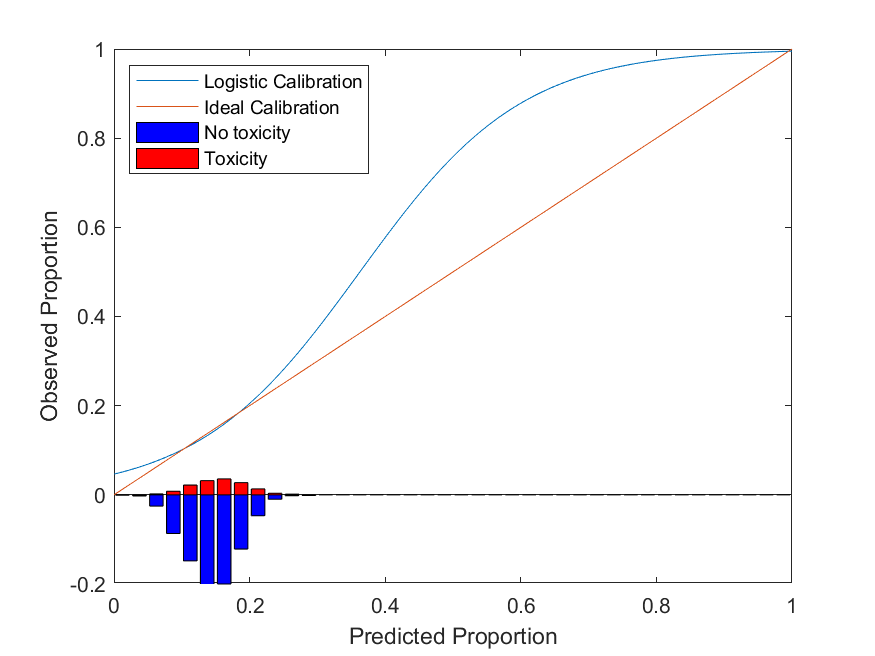


## Figure 2A. Rectal Bleeding G2+ Binned Calibration Plot: LKB-EQD2 Model


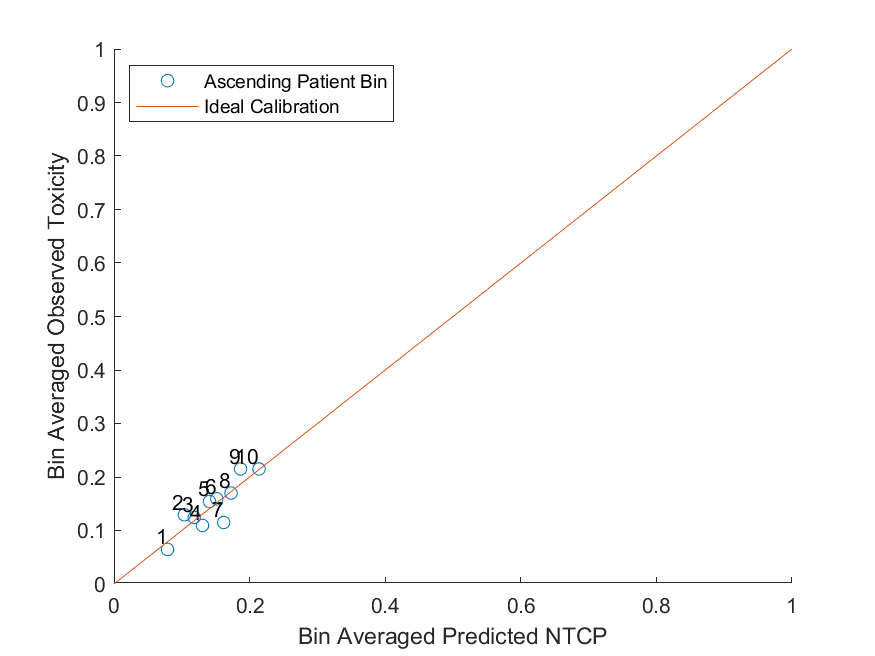


## Figure 3A. Stool Frequency G1+ Calibration Curve: LKB-EQD2 Model


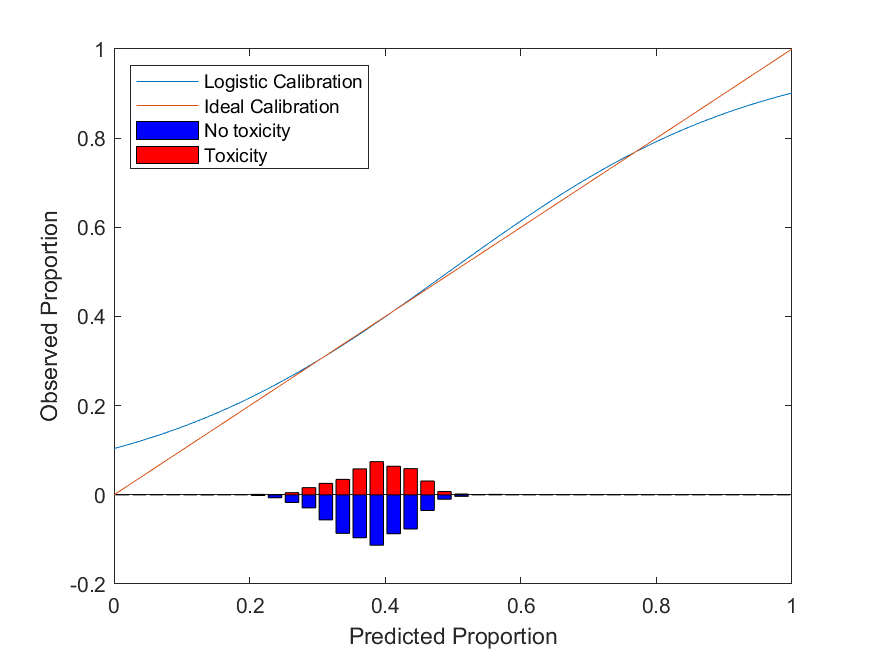


## Figure 4A. Stool Frequency G1+ Binned Calibration Plot: LKB-EQD2 Model


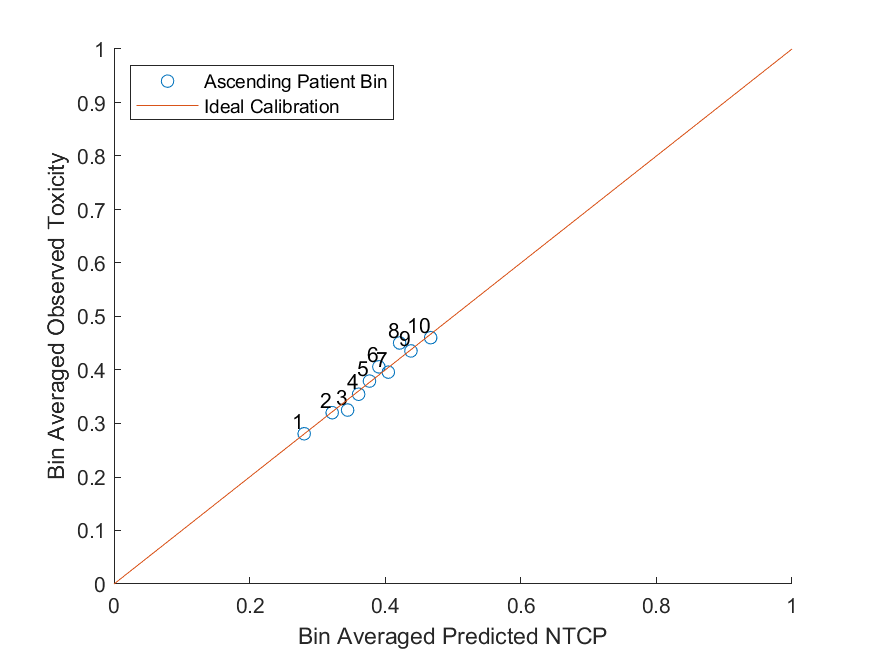


## Figure 5A. Stool Frequency G2+ Calibration Curve: LKB-EQD2 Model


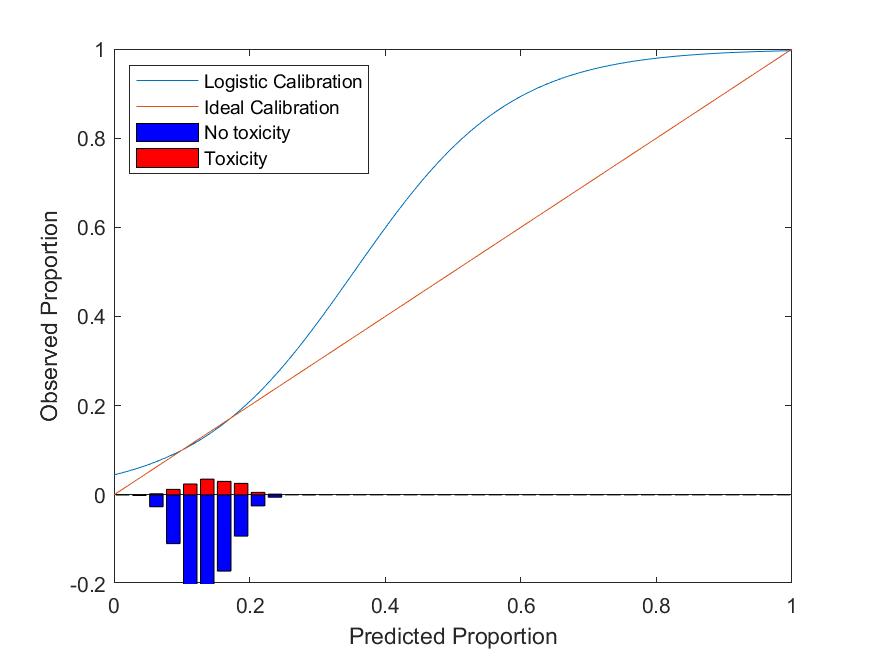


## Figure 6A. Stool Frequency G2+ Binned Calibration Plot: LKB-EQD2 Model


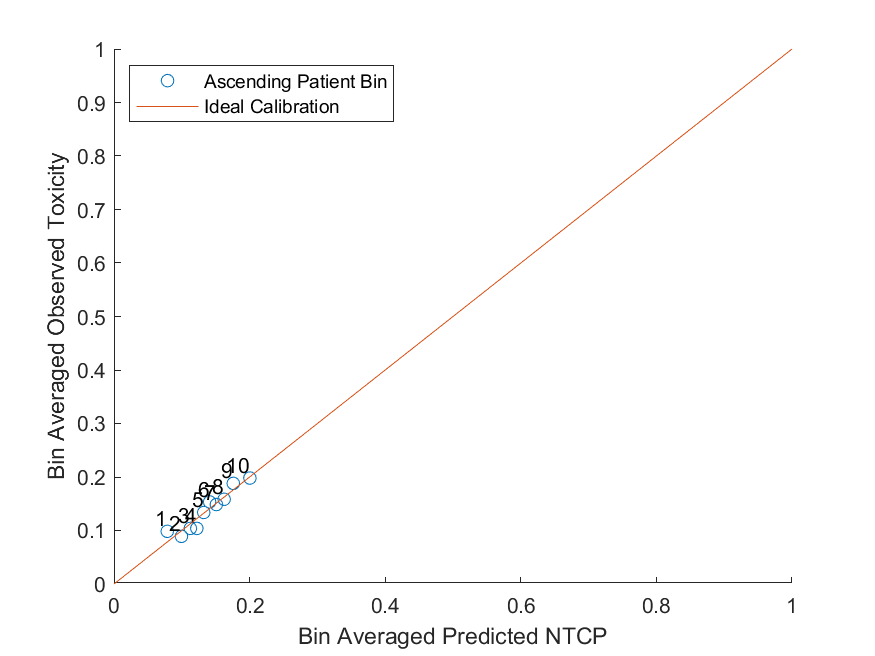


## Figure 7A. Pain G1+ Calibration Curve: LKB-EQD2 Model


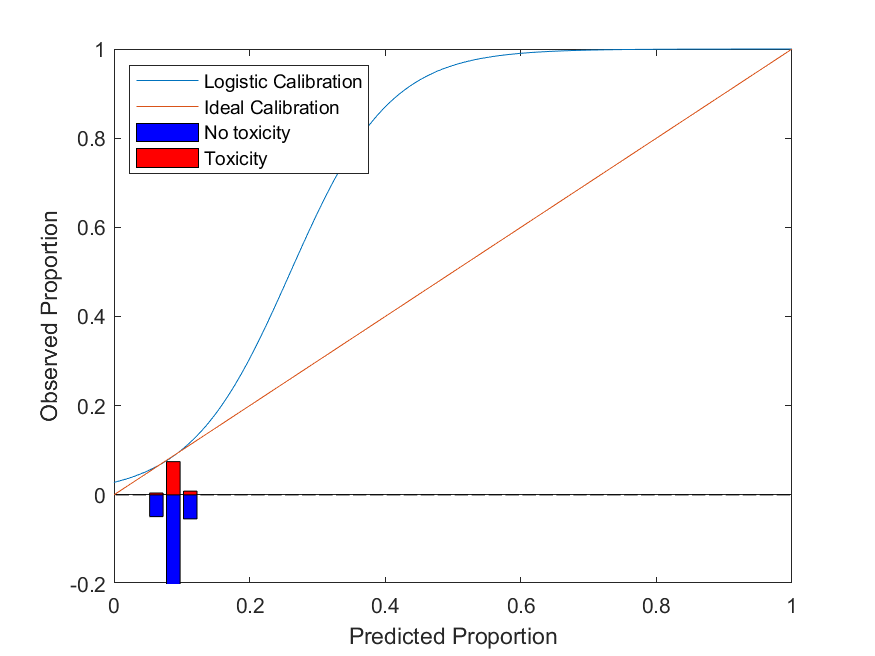


## Figure 8A. Pain G1+ Binned Calibration Plot: LKB-EQD2 Model


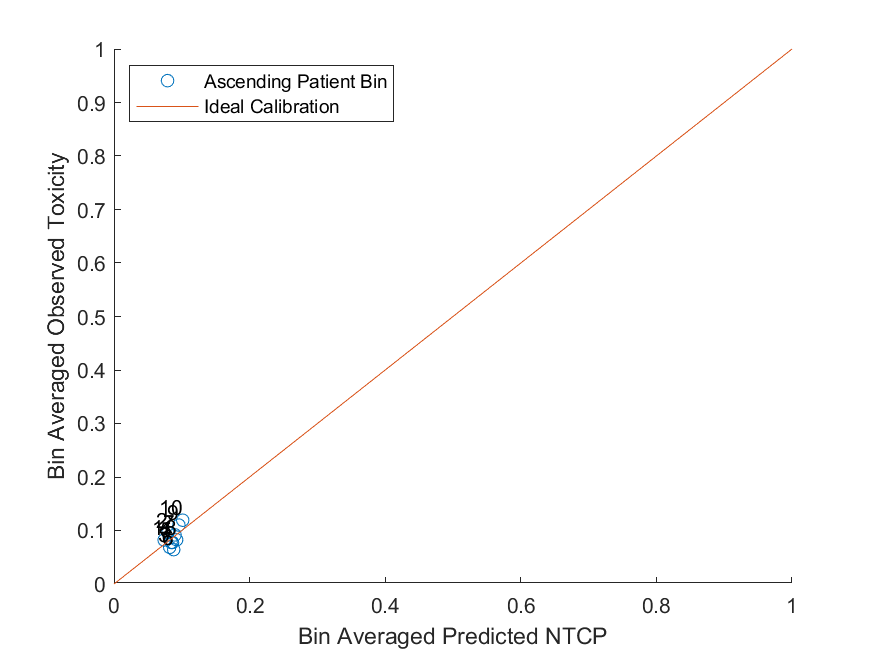


## Figure 9A. Proctitis G1+ Calibration Curve: LKB-EQD2 Model


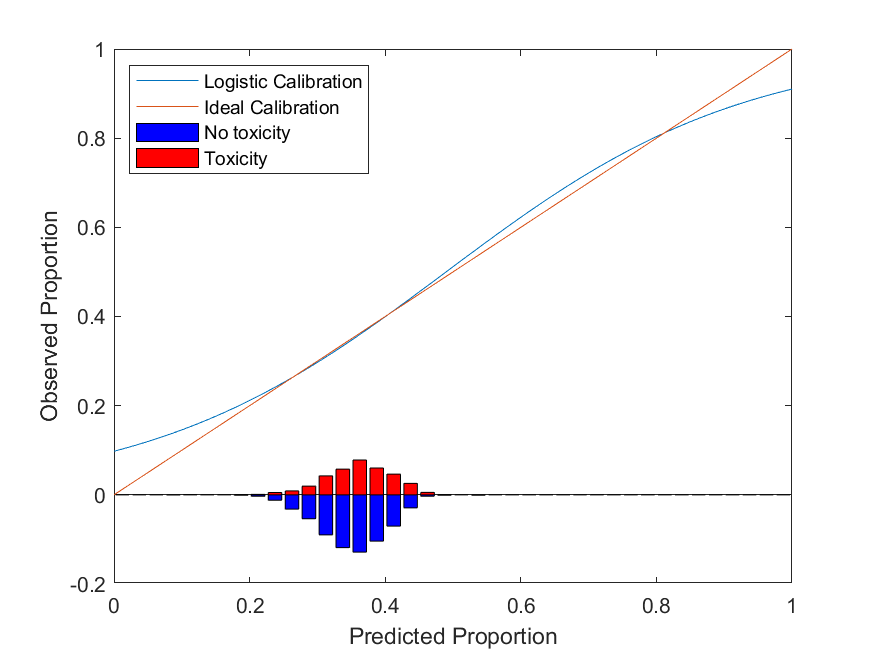


## Figure 10A. Proctitis G1+ Binned Calibration Plot: LKB-EQD2 Model


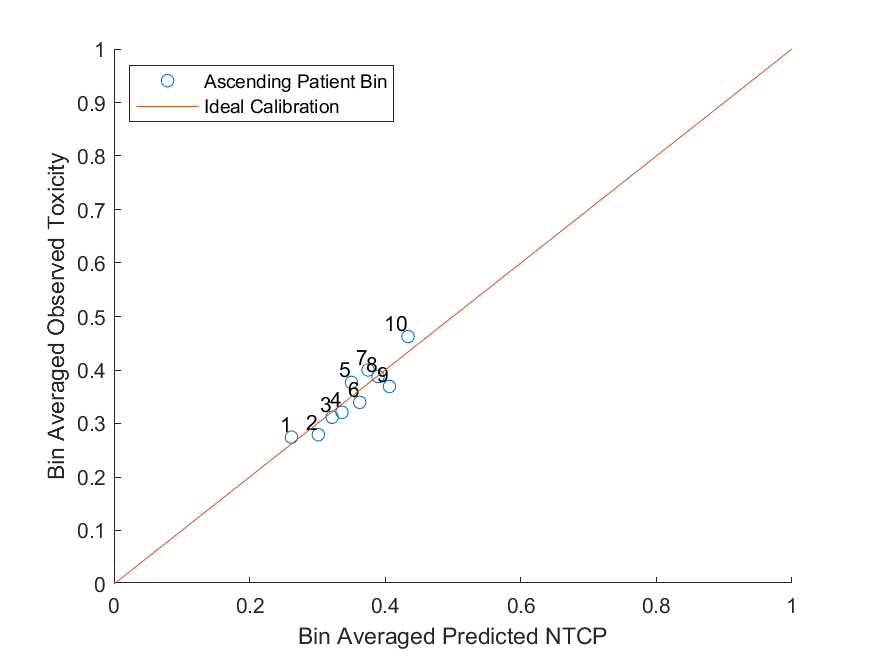


## Figure 11A. Proctitis G2+ Calibration Curve: LKB-EQD2 Model


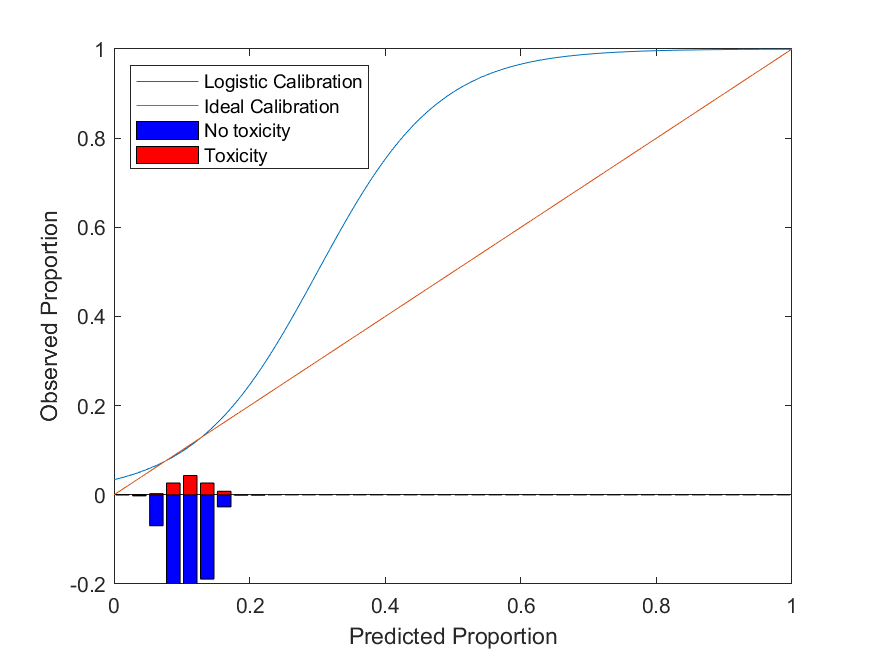


## Figure 12A. Proctitis G2+ Binned Calibration Plot: LKB-EQD2 Model


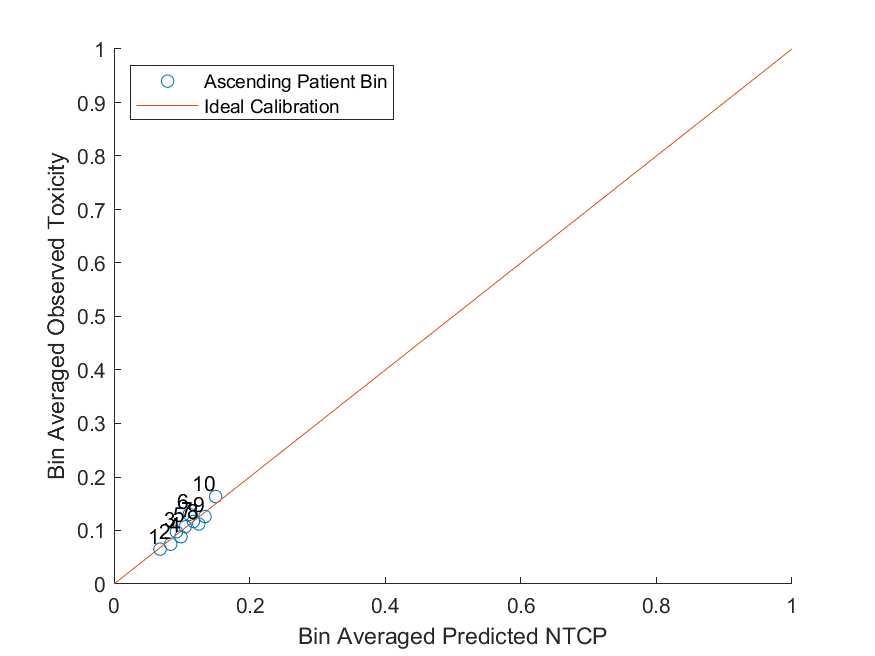


## Figure 13A. Sphincter Control G1+ Calibration Curve: LKB-EQD2 Model


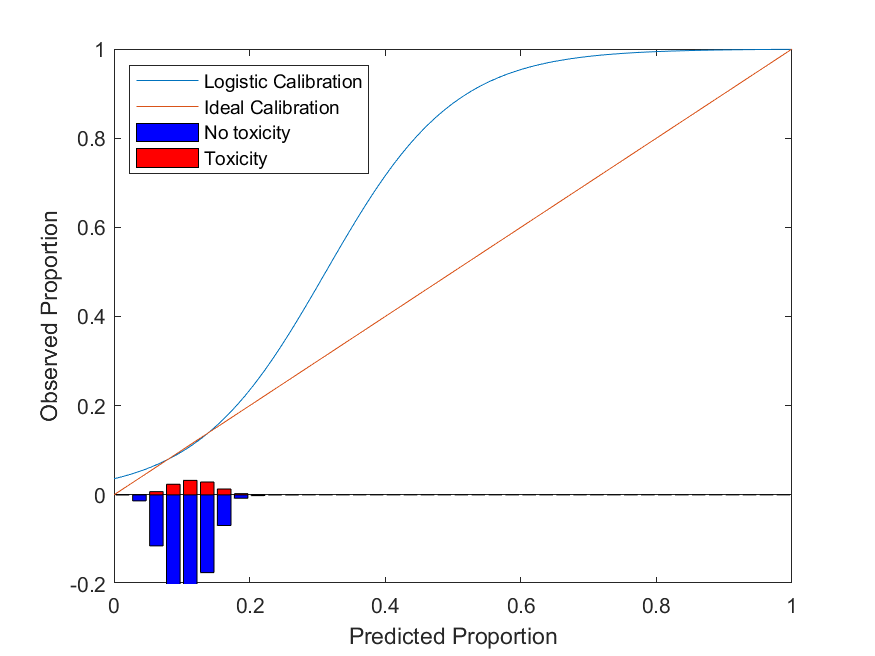


## Figure 14A. Sphincter Control G1+ Binned Calibration Plot: LKB-EQD2 Model


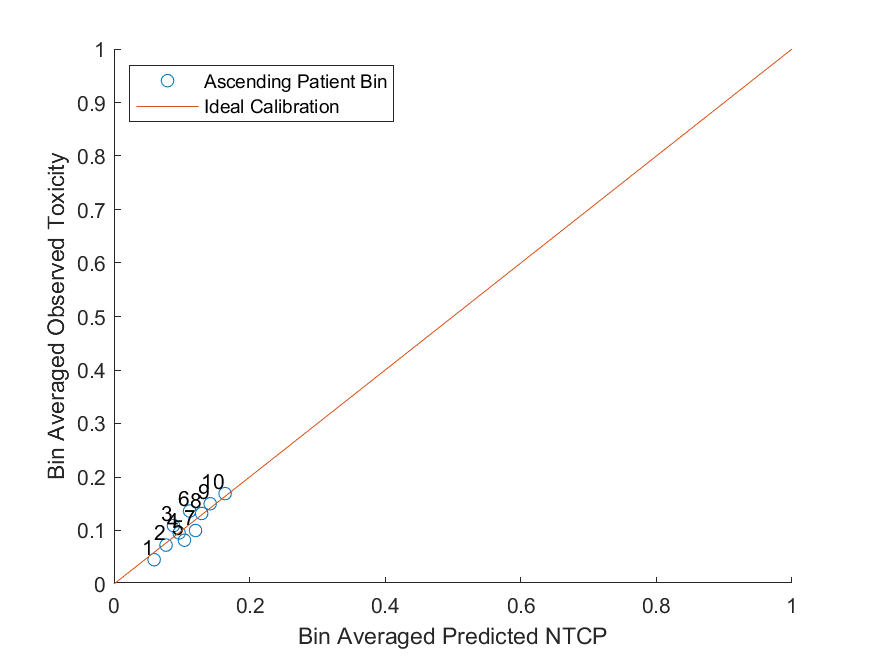


## Figure 15A. Stricture/Ulcer G1+ Calibration Curve: LKB-EQD2 Model


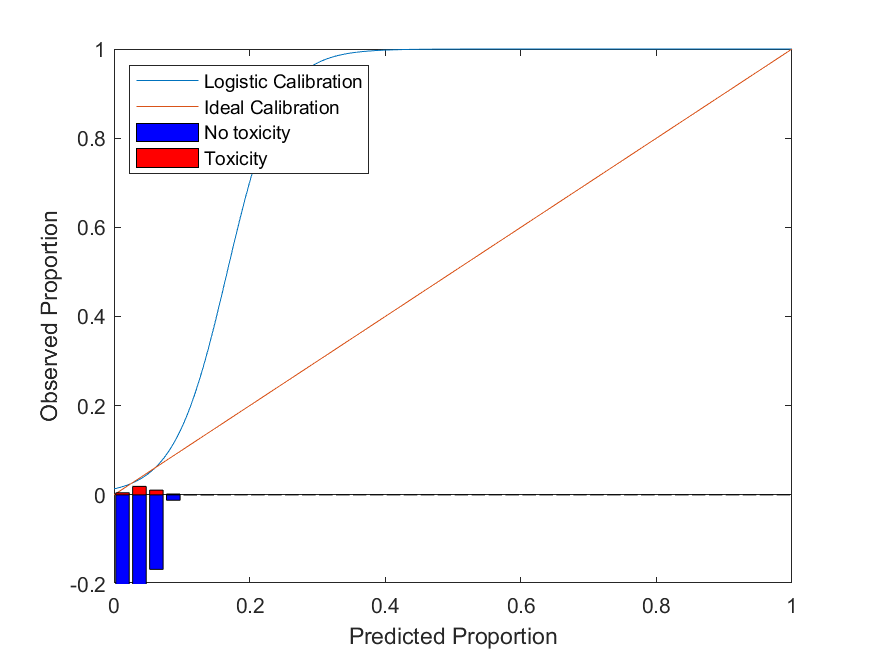


## Figure 16A. Stricture/Ulcer G1+ Binned Calibration Plot: LKB-EQD2 Model


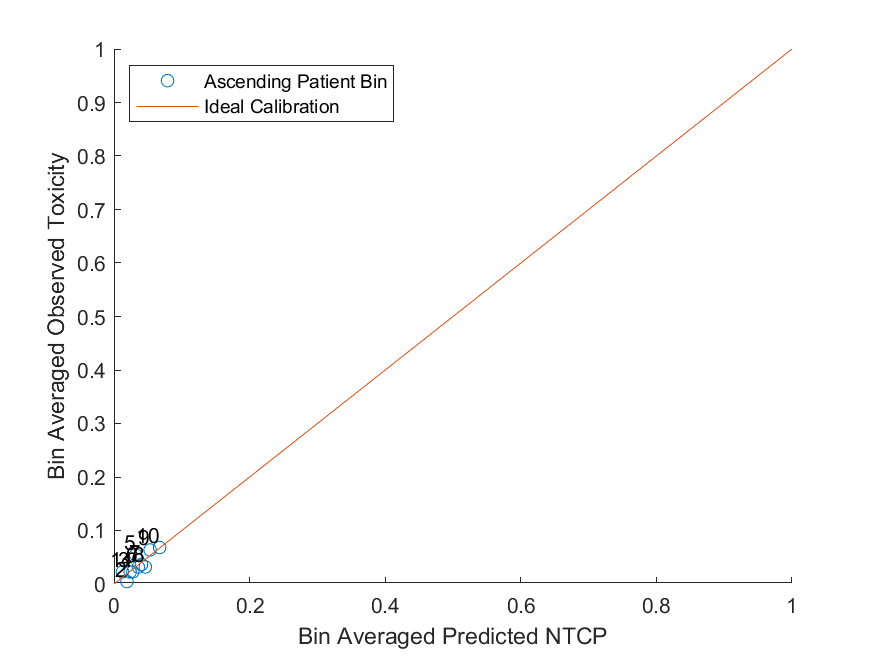


# Appendix D. Calibration Plots for LKB-EQD2-DMF Models Significantly Improving on LKB-EQD2 Model

## Figure 17A. Stool Frequency G2+ Calibration Curve: LKB-EQD2-DMF Model (DMF = IBD/Diverticular)


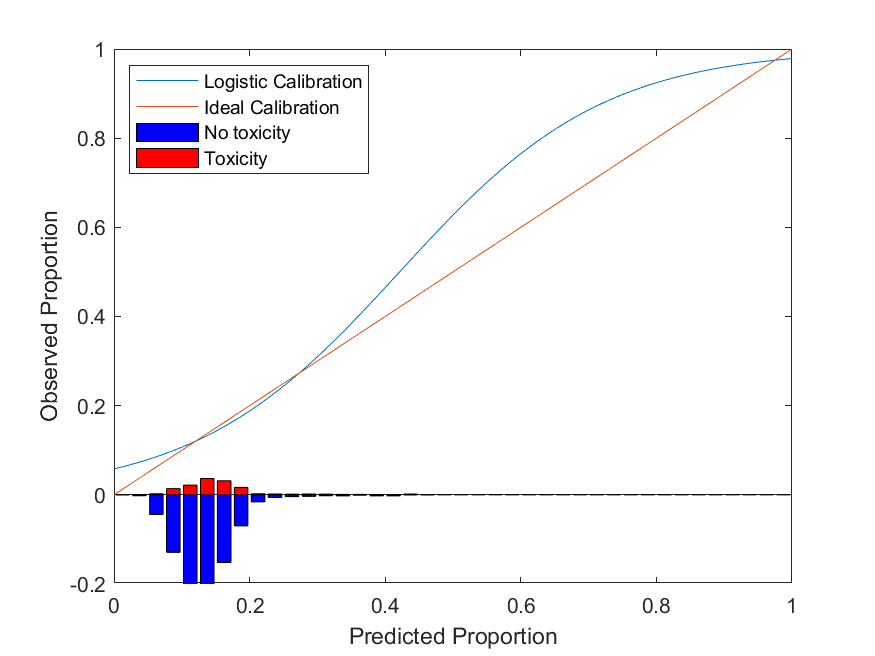


## Figure 18A. Stool Frequency G2+ Binned Calibration Plot: LKB-EQD2-DMF Model (DMF = IBD/Diverticular)


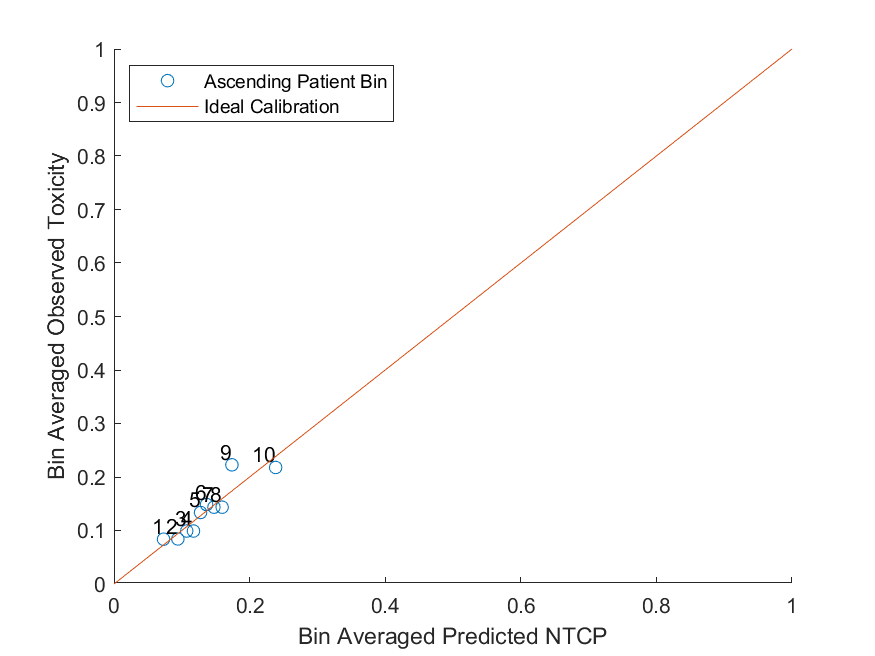


## Figure 19A. Proctitis G1+ Calibration Curve: LKB-EQD2-DMF Model (DMF = IBD/Diverticular)


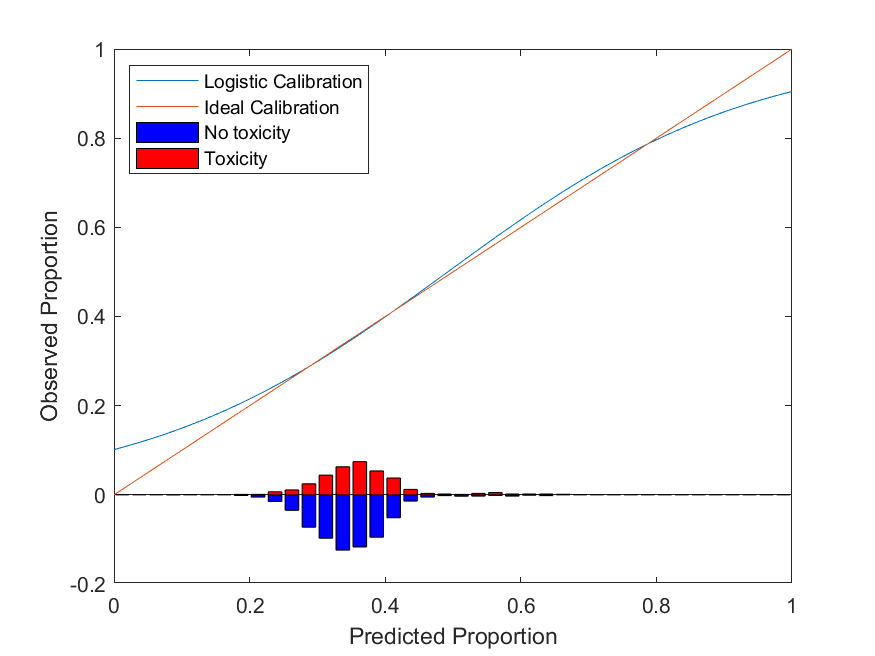


## Figure 20A. Proctitis G1+ Binned Calibration Plot: LKB-EQD2-DMF Model (DMF = IBD/Diverticular)


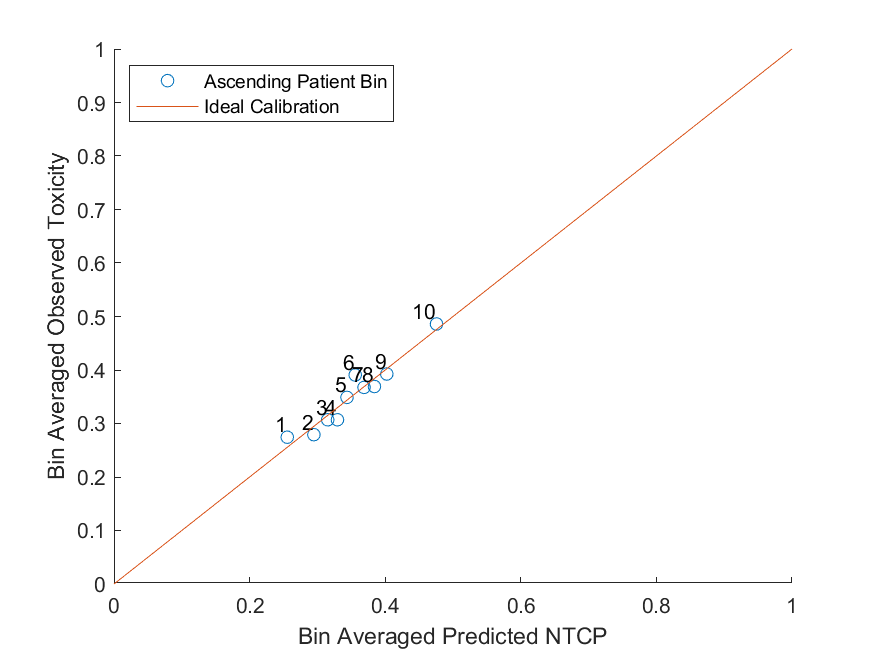


# Table 3A. Calculation of Pooled Rectal Late α/β Ratio

Calculation for the pooled averaged late rectal α/β ratio for those more common endpoints fitted best during modelling. Weighting is by the frequency of side effect occurrence seen in patients modelled (per **Table 2**).

| **Late Rectal Endpoints** | **Frequency** | **Weights** | **α/β Ratio (Gy)** |
| --- | --- | --- | --- |
| **Grade 1+ Endpoints** | | | |
| Bleeding G1+ | 0.329 | 9.139 | 1.58 |
| Frequency G1+ | 0.381 | 10.583 | 2.26 |
| Pain G1+ | 0.087 | 2.417 | 3.64 |
| Proctitis G1+ | 0.352 | 9.778 | 2.65 |
| Sphincter Control G1+ | 0.109 | 3.028 | 3.09 |
| Stricture/Ulcer G1+ | 0.036 | 1 | 2.49 |
| **Grade 1+ weighted average** | | | **2.36** |
|  | | | |
| **Grade 2+ Endpoints** | | | |
| Bleeding G2+ | 0.146 | 1.352 | 1.71 |
| Frequency G2+ | 0.138 | 1.278 | 2.66 |
| Proctitis G2+ | 0.108 | 1 | 2.70 |
| **Grade 2 weighted average** | | | **2.32** |

# Table 4A. LKB-NoEQD2 Parameter Comparison

Parameters n, m, TD50 for LKB model without EQD2 correction fitted on conventionally fractionated 74Gy Patients. Comparing with other studies fitting similar endpoints. Defraene and Peeters incontinence data omitted as modelled only on anal wall OAR.

| **Endpoint** | **Study** | **Pts** | **n** | **95% CI**  *(68% CI)* | **m** | **95% CI**  *(68% CI)* | **TD50** | **95% CI**  *(68% CI)* |
| --- | --- | --- | --- | --- | --- | --- | --- | --- |
| Bleeding G1+ | This Study | 644 | 0.26 | 0.01-1.12 | 0.33 | 0.09–0.68 | 61.5 | 54.5–74.0 |
|  | Gulliford *et al* [1] | 361 | 0.14 | 0.09–0.16 | 0.26 | 0.18–0.48 | 59.2 | 57.8–61.9 |
| Bleeding G2+ | This Study | 642 | 0.13 | 0.01–0.42 | 0.21 | 0.06–0.43 | 74.0 | 67.2–96.6 |
|  | Gulliford *et al* | 361 | 0.12 | 0.10–0.16 | 0.14 | 0.12–0.16 | 68.2 | 64.9–69.3 |
|  | Peeters *et al* [2] | 468 | 0.13 | *(0.04–0.25)* | 0.14 | *(0.11–0.19)* | 81.0 | *(75–90)* |
|  | Defraene *et al* [3] * | 512 | 0.18 | *(0.09–0.33)* | 0.15 | *(0.12–0.20)* | 79.0 | *(74.0–86.5)* |
|  | Rancati *et al* [4] | 547 | 0.23 | *(0.14–0.42)* | 0.19 | *(0.15–0.25)* | 81.9 | *(76.8–91.2)* |
| Frequency G1+ | This Study | 643 | 0.17 | 0.01–0.53 | 0.30 | 0.09–0.76 | 60.8 | 53.7–72.8 |
|  | Gulliford *et al* | 344 | 0.30 | 0.16–0.6 | 0.60 | 0.41–>1 | 61.5 | 56.3–68.3 |
| Frequency G2+ | This Study | 642 | 0.11 | 0.03–0.69 | 0.20 | 0.09–0.49 | 73.8 | 66.2–98.6 |
|  | Peeters *et al* | 468 | 0.39 | *(0.19–1.11)* | 0.24 | *(0.18–0.35)* | 84.0 | *(75–103)* |
|  | Defraene *et al* * | 512 | 1.18 | *(0.94–1.53)* | 0.34 | *(0.27–0.44)* | 97.4 | *(82.4–137.5)* |
| Proctitis G1+ | This Study | 691 | 0.10 | 0.01–0.18 | 0.22 | 0.08–0.50 | 64.9 | 60.8–73.7 |
|  | Gulliford *et al* | 388 | 0.14 | 0.11–0.20 | 0.28 | 0.19–0.60 | 58.2 | 55.7–60.1 |
| Proctitis G2+ | This Study | 691 | 0.05 | 0.01–0.14 | 0.14 | 0.06–0.44 | 78.0 | 71.6-111.6 |
|  | Gulliford *et al* | 388 | 0.15 | 0.11–0.20 | 0.20 | 0.19–0.24 | 67.0 | 64.8–69.3 |

* Rectal wall instead of solid rectum.

# Table 5A. Moderate Hypofractionation Trial Design Assumptions

PACE-B not included as late toxicity not reported, however late rectal α/β ratio was assumed to be 3 Gy in that trial [5]. Trial references are the same as those in **Table 5A**.

Abbreviations: EQD2 = Equivalent Dose in 2 Gy Fractions.

| Trial | Prostate Assumptions | | | | Rectum Assumptions | | | | Design | |
| --- | --- | --- | --- | --- | --- | --- | --- | --- | --- | --- |
|  | α/β  Ratio | Test  EQD2 | Control  EQD2 | α/β  Ratio | | Test  EQD2 | Control  EQD2 |  | |  |
| CHHiP 57Gy | 1.5 - 2.5 | 73.3 – 69.7 | 74 | 3 | | 68.4 | 74 | Isoeffective | |  |
| CHHiP 60Gy | 1.5 - 2.5 | 77.1 – 73.3 | 74 | 3 | | 72 | 74 | Isoeffective | |  |
| PROFIT | 1 - 3 | 80 - 72 | 78 | 3 - 5 | | 72 - 68.6 | 78 | Isoeffective | |  |
| RTOG 0415 | 3 | 77 | 70.8 | 3 | | 77 | 70.8 | Dose Escalation | |  |
| HYPRO | 1.5 | 90.4 | 78 | 4 - 6 | | 79.7 - 76 | 78 | Isotoxic | |  |
| HYPO-RT-PC | <3 | >78 | 78 | 3 | | 77.7 | 78 | Isotoxic | |  |

# Table 6A. Bowel Toxicity in Phase III Hypofractionation Trials

| **Trial** | **Patients** | **Treatment Arms** | **Timepoint for Toxicity** | **Cumulative RTOG Late Bowel Toxicity** | |
| --- | --- | --- | --- | --- | --- |
|  | **n** | **Gy / Fractions / Weeks** |  | **G2+** | **G3+** |
| **CHHiP** [6] | 1065  1074  1077 | C 74 Gy / 37 Fr / 7.4 w  H 60 Gy / 20 Fr / 4.0 w  H 57 Gy / 19 Fr / 3.8 w | 5 years median | 13.7%  11.9%  11.3% | 0%  <1%  <1% |
| **PROFIT** [7] | 598  608 | C 78 Gy / 39 Fr / 7.8 w  H 60 Gy / 20 Fr / 4.0 w | 6 years median | 13.9%  8.9% | 2.9%  1.5% |
| **RTOG 0415** [8] | 558  557 | C 73.8 Gy / 41 Fr / 8.2 w  H 70 Gy / 28 Fr / 5.6 w | 5.8 years median | 14.0%  22.4% | 2.6%  4.1% |
| **HYPRO** [9] | 410  410 | C 78 Gy / 39 Fr / 7.8w  H 64.6 Gy / 19 Fr / 6.5w | At 3 years | 17.7%  21.9% | 2.6%  3.3% |
| **HYPO-RT-PC** [10] | 602  598 | C 78 Gy / 39 Fr / 7.8w  H 42.7 Gy / 7 Fr / 2.5w | 5 years  median | 9.7%  9.5% | 1.9%  1.5% |

**Legend**

Table 1. Summary of the phase III trials of hypofractionated radiotherapy for localised prostate cancer, with reference to subsequent late bowel toxicity. PACE-B not included, as late toxicity not yet reported.

RTOG = Radiation Therapy Oncology Group

GX+ = Grade X toxicity or worse. Fr = Fractions. w = Weeks over which treatment delivered

# Bibliography for Supplementary Appendix

[1] Gulliford SL, Partridge M, Sydes MR, Webb S, Evans PM, Dearnaley DP. Parameters for the Lyman Kutcher Burman (LKB) model of Normal Tissue Complication Probability (NTCP) for specific rectal complications observed in clinical practise. Radiother Oncol 2012;102:347–51. doi:10.1016/j.radonc.2011.10.022.

[2] Peeters STH, Hoogeman MS, Heemsbergen WD, Hart AAM, Koper PCM, Lebesque J V. Rectal bleeding, fecal incontinence, and high stool frequency after conformal radiotherapy for prostate cancer: Normal tissue complication probability modeling. Int J Radiat Oncol Biol Phys 2006;66:11–9. doi:10.1016/j.ijrobp.2006.03.034.

[3] Defraene G, Van Den Bergh L, Al-Mamgani A, Haustermans K, Heemsbergen W, Van Den Heuvel F, et al. The benefits of including clinical factors in rectal normal tissue complication probability modeling after radiotherapy for prostate cancer. Int J Radiat Oncol Biol Phys 2012;82:1233–42. doi:10.1016/j.ijrobp.2011.03.056.

[4] Rancati T, Fiorino C, Gagliardi G, Cattaneo GM, Sanguineti G, Borca VC, et al. Fitting late rectal bleeding data using different NTCP models: Results from an Italian multi-centric study (AIROPROS0101). Radiother Oncol 2004;73:21–32. doi:10.1016/j.radonc.2004.08.013.

[5] Brand DH, Tree AC, Ostler P, van der Voet H, Loblaw A, Chu W, et al. Intensity-modulated fractionated radiotherapy versus stereotactic body radiotherapy for prostate cancer (PACE-B): acute toxicity findings from an international, randomised, open-label, phase 3, non-inferiority trial. Lancet Oncol 2019;20:1531–43. doi:10.1016/S1470-2045(19)30569-8.

[6] Dearnaley D, Syndikus I, Mossop H, Khoo V, Birtle A, Bloomfield D, et al. Conventional versus hypofractionated high-dose intensity-modulated radiotherapy for prostate cancer: 5-year outcomes of the randomised, non-inferiority, phase 3 CHHiP trial. Lancet Oncol 2016;17:1047–60. doi:10.1016/S1470-2045(16)30102-4.

[7] Catton CN, Lukka H, Gu C-S, Martin JM, Supiot S, Chung PWM, et al. Randomized Trial of a Hypofractionated Radiation Regimen for the Treatment of Localized Prostate Cancer. J Clin Oncol 2017;35:1884–90. doi:10.1200/JCO.2016.71.7397.

[8] Lee WR, Dignam JJ, Amin MB, Bruner DW, Low D, Swanson GP, et al. Randomized phase III noninferiority study comparing two radiotherapy fractionation schedules in patients with low-risk prostate cancer. J Clin Oncol 2016;34:2325–32. doi:10.1200/JCO.2016.67.0448.

[9] Incrocci L, Wortel RC, Alemayehu WG, Aluwini S, Schimmel E, Krol S, et al. Hypofractionated versus conventionally fractionated radiotherapy for patients with localised prostate cancer (HYPRO): final efficacy results from a randomised, multicentre, open-label, phase 3 trial. Lancet Oncol 2016;17:1061–9. doi:10.1016/S1470-2045(16)30070-5.

[10] Widmark A, Gunnlaugsson A, Beckman L, Thellenberg-Karlsson C, Hoyer M, Lagerlund M, et al. Ultra-hypofractionated versus conventionally fractionated radiotherapy for prostate cancer: 5-year outcomes of the HYPO-RT-PC randomised, non-inferiority, phase 3 trial. Lancet 2019;394:385–95. doi:10.1016/S0140-6736(19)31131-6.
